# RAS signalling through PI3-Kinase controls cell migration via modulation of Reelin expression

**DOI:** 10.1038/ncomms11245

**Published:** 2016-04-13

**Authors:** Esther Castellano, Miriam Molina-Arcas, Agata Adelajda Krygowska, Philip East, Patricia Warne, Alastair Nicol, Julian Downward

**Affiliations:** 1 Oncogene Biology, The Francis Crick Institute, 44 Lincoln's Inn Fields, London WC2A 3LY, UK; 2 Centre for Cancer and Inflammation, Barts Cancer Institute, Queen Mary University of London, London EC1M 6BQ, UK; 3 Lung Cancer Group, The Institute of Cancer Research, 237 Fulham Road, London SW3 6JB, UK; 4 Computational Biology, The Francis Crick Institute, 44 Lincoln's Inn Fields, London WC2A 3LY, UK; 5 Light Microscopy Laboratories, The Francis Crick Institute, 44 Lincoln's Inn Fields, London WC2A 3LY, UK

## Abstract

RAS signalling through phosphoinositide 3-kinase (PI3-Kinase) has been shown to have an essential role in tumour initiation and maintenance. RAS also regulates cell motility and tumour invasiveness, but the role of direct RAS binding to PI3-Kinase in this remains uncertain. Here, we provide evidence that disruption of RAS interaction with PI3-Kinase p110α decreases cell motility and prevents activation of Rac GTPase. Analysis of gene expression in cells lacking RAS interaction with p110α reveals increased levels of the extracellular matrix glycoprotein Reelin and activation of its downstream pathway resulting in upregulation of E-cadherin expression. Induction of the Reelin/E-cadherin axis is also observed in Kras mutant lung tumours that are regressing due to blockade of RAS interaction with PI3-Kinase. Furthermore, loss of Reelin correlates with decreased survival of lung and breast cancer patients. Reelin thus plays a role in restraining RAS and PI3-kinase promotion of cell motility and potentially tumour metastasis.

## Abstract (web summary)

Ras signalling through PI3K kinase has an important role in tumour initiation and progression. Here, the authors show that the interaction of Ras with PI3-Kinase p110α and the subsequent activation of Rac-GTPase impairs cell -cell interaction by blocking the downstream Reelin/E-cadherin, thus resulting in cell migration.

## Graphical abstract

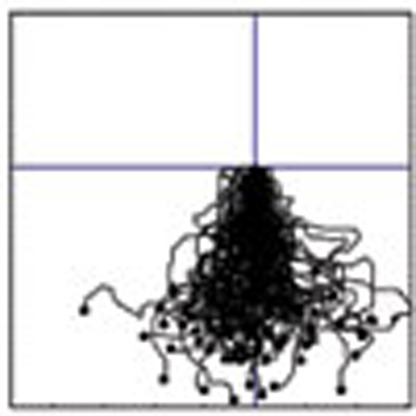

Cell migration is a complex highly coordinated process that is essential for many diverse biological processes in animals, including embryonic morphogenesis, immune surveillance, tissue homoeostasis and wound healing. Errors during this process have important consequences, including mental retardation, vascular disease, tumour formation and metastasis. A better understanding of the mechanisms by which cells migrate may lead to the development of novel therapeutic strategies for controlling the invasive behaviour of tumour cells^1^,^2^,^3^.

Acquisition of an increased migratory phenotype, accompanied by extensive remodelling of the actin cytoskeleton is one of the first requirements in metastasis formation. Oncogenic activation of RAS has been implicated in facilitating almost all aspects of the malignant phenotype^4^,^5^. Oncogenic RAS contributes to this process by inducing alterations in cell–cell and cell–matrix interactions, and the acquisition of a migratory phenotype. The perturbation of cell–cell contacts by oncogenic RAS is accomplished through the targeting of the molecular machinery that maintains intercellular adhesion junctions, including the E-cadherin receptor and its associated cytoplasmic protein β-catenin^6^,^7^. Also, oncogenic RAS directly contributes to the enhanced motility of cancer cells by affecting pronounced changes in the polymerization, organization and contraction of actin; the polymerization and/or stability of microtubules; and the transcriptional regulation of mitogenic gene products^4^,^8^. Collectively, these changes establish the front-rear asymmetry that is required for cell migration.

Although a significant number of studies have analysed the role of Rho family GTPase signalling pathways in RAS-induced transformation, relatively little is known about the differential regulation of Rho GTPases by RAS oncogenes, or their subsequent contribution to oncogene-specific cell migration properties. It is well known that extracellular signal-regulated kinase signalling is important for cell motility through Rho GTPases^8^,^9^. The PI3-Kinase pathway is also involved in Rho family signal transduction, affects cell migration^10^,^11^ and has been implicated in metastasis of RAS mutant lung tumours^12^. Oncogenic RAS is required for both induction and maintenance of epithelial to mesenchymal transition, mainly through its downstream effector extracellular signal-regulated kinase and increased cell migration and invasion mediated by Rac1 (refs 13, 14, 15). However, the specific role that RAS plays in tumour invasion and metastasis or the main effector pathways through which RAS contributes to metastasis formation are still poorly understood. Defining the precise modes by which RAS-responsive pathways affect metastatic capacity awaits an improved understanding of the context-dependent outcome of their coordinated activation.

In this study, we undertook an analysis of the migration of mouse embryo fibroblasts derived from a mouse model in which RAS cannot interact with PI3-Kinase due to the introduction of two point mutations (T208D and K227A) in the RAS-binding domain (RBD) of the endogenous *Pik3ca* gene^16^. Our experimental data show that RAS, through its interaction with PI3-Kinase, regulates migration of cells in response to several growth factors by regulating Rac activation. We also identify a key role for Reelin (RELN) as a regulator of cell migration downstream of RAS-PI3-Kinase signalling and show that this interaction controls Reelin messenger RNA (mRNA) stability, thus regulating its expression. Activation of the Reelin downstream pathway prevents cells from migrating and results in the upregulation of E-cadherin, thereby impacting on cell–cell interaction. These results provide a better understanding of how PI3-Kinase signalling contributes to RAS-driven invasiveness and metastatic potential and may lead to the development of more effective therapies that prevent metastatic spread of primary tumours.

## Results

### Disruption of RAS-PI3-Kinase binding impairs cell motility

We have previously reported the generation of a mouse model in which the interaction between RAS and PI3-Kinase is disrupted by two point mutations on the RBD of the endogenous *Pik3ca* gene (T208D and K227A)^16^. Here we use mouse embryonic fibroblasts (MEFs) isolated from Pik3ca^RBD^ mice to investigate how signalling through the RAS-PI3-Kinase axis regulates cell motility.

**Figure 1:**
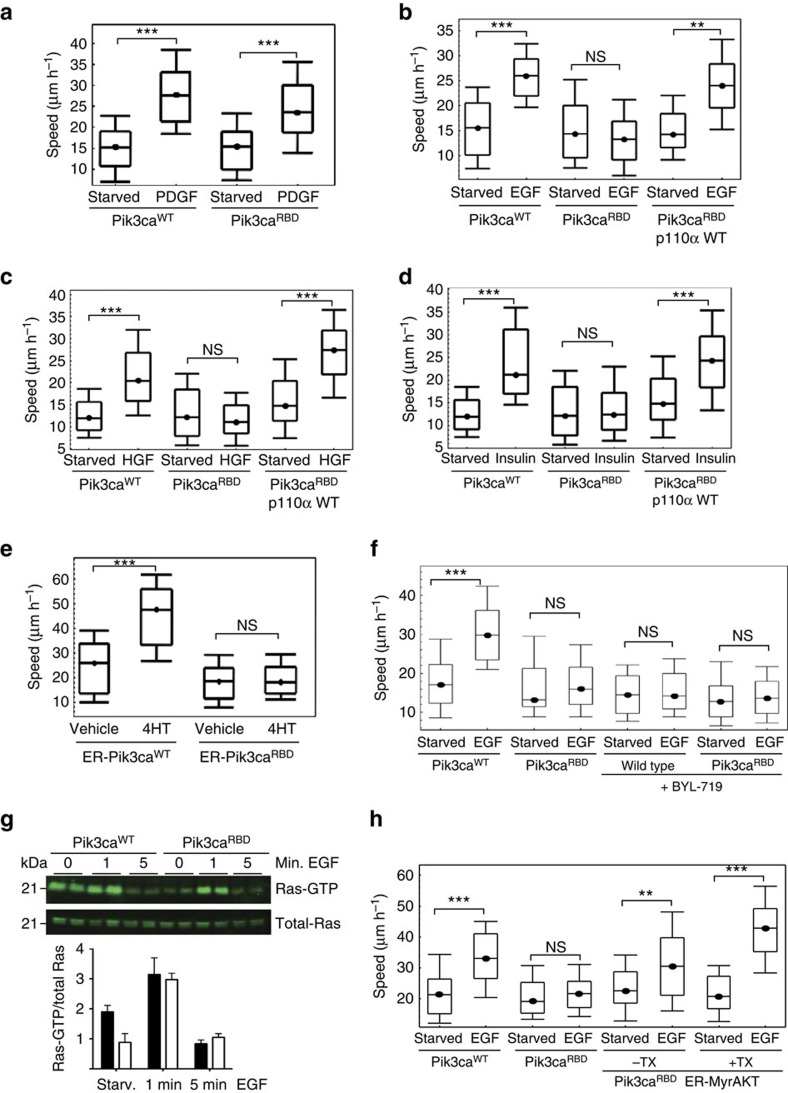
Removal of RAS Interaction with p110α impairs cell motility. (**a**–**d**) Random migration of Pik3ca^WT^, Pik3ca^RBD^ and Pik3ca^RBD^ containing a WT p110a was analysed by time-lapse video microscopy and cell tracing in the presence or absence of (**a**) PDGF (20 ng ml^−1^); (**b**) EGF (20 ng ml^−1^); (**c**) HGF (10 ng ml^−1^); (**d**) Insulin (100 ng ml^−1^). Cells were imaged at 10 min intervals for 18 h. Graphs show migration tracks obtained from 90 cells in each experimental condition. The data are represented as a box and whisker plot in which the box shows the interquartile range that contains values between 25th and 75th percentile. The line inside the box shows the median. The two whiskers show adjacent values. The upper adjacent value (upper mark) is the value of the largest observation that is less than or equal to the upper quartile plus 1.5 the length of the interquartile range. Analogously the lower adjacent value (lower mark) is the value of the smallest observation that is greater than or equal to the lower quartile less 1.5 times the length of interquartile range. Analysis of variance (ANOVA) statistical analysis was performed with starved cells used as reference for each condition (NS, not significant; **P<0.01; ***P<0.001). (**e**) Random migration assays of Pik3ca^WT^ and Pik3ca^RBD^ cells containing ER-RAS V12 (tamoxifen-inducible H-RAS V12) treated with 4-hydroxytamoxifen (TX) or vehicle control. ANOVA statistical analysis was performed with starved cells used as reference for each condition (NS, not significant; ***P<0.001). Box and whisker plot was generated as indicated for **a**. (**f**) Random migration of Pik3ca^WT^ and Pik3ca^RBD^ cells was analysed by time-lapse video microscopy and cell tracing in the presence or absence of EGF (20 ng ml^−1^) and the p110α specific PI3-kinase inhibitor BYL-719 (500 nM). Assay was carried out as described for **a**. ANOVA statistical analysis was performed with starved cells used as reference for each condition (NS, not significant; ***P<0.001). Box and whisker plot was generated as indicated for **a**. (**g**) Pik3ca^WT^ (filled bars) and Pik3ca^RBD^ (empty bars) MEFs were stimulated with EGF (20 ng ml^−1^) for the indicated time periods. RAS-GTP activity was established in pull-down assays using GST-RBD of Raf (GST-RafRBD). Both total lysates and proteins bound to GST-RafRBD were analysed by western blot to detect RAS. Lower panel, quantitation of pull-down assays. (**h**) Random migration of Pik3ca^WT^, Pik3ca^RBD^ and Pik3ca^RBD^ cells containing ER-MyrAkt was analysed by time-lapse video microscopy and cell tracing in the presence or absence EGF (20 ng ml^−1^) and 4-hydroxytamoxifen (TX). ANOVA statistical analysis was performed with starved cells used as reference for each condition (NS, not significant; **P<0.01; ***P<0.001). Box and whisker plot was generated as indicated for **a**.

First, we explored whether the disruption of the RAS-PI3-Kinase interaction affected cell migration. The increase in migration speed observed in response to foetal bovine serum (FBS), platelet derived growth factor (PDGF) or fibroblast growth factor 2 (FGF2) stimulation was similar in Pik3ca^WT^ and Pik3ca^RBD^ MEFs (Fig. 1a, and Supplementary Fig. 1a,b). However, the migration speed of the Pik3ca^RBD^ MEFs was not increased by epidermal growth factor (EGF) (Fig. 1b), hepatocyte growth factor (HGF) (Fig. 1c) or insulin treatment (Fig. 1d), although these were effective on Pik3ca^WT^ cells. In all cases, the increase in migration speed was restored on introduction of a wild type (WT) p110α to cells, confirming that the defect was due to the disruption in RAS-PI3-Kinase signalling.

We also investigated whether the RBD mutation had an impact on the migration speed in response to oncogenic RAS (HRAS V12) expression, using a tamoxifen-inducible fusion protein of activated RAS with the hormone-binding domain of the oestrogen receptor. Although induction of oncogenic RAS had a great impact on the migration of Pik3ca^WT^ cells, Pik3ca^RBD^ cells were not affected and migration was not increased (Fig. 1e). Similar results were obtained when a constitutively active oncogenic RAS was expressed (Supplementary Fig. 1c).

To confirm that the migration phenotype of Pik3ca^RBD^ cells treated with EGF was due to a defect in PI3-Kinase signalling, we investigated the effect of treating both WT and RBD-mutant cells with a specific PI3-Kinase p110α inhibitor (BYL-719) or with a pan-PI3-Kinase inhibitor (PI-103). Both inhibitors led to abrogation of the effect of EGF on migration of Pik3ca^WT^ MEFs (Fig. 1f and Supplementary Fig. 1d), but had no additional effect in migration of Pik3ca^RBD^ cells. MAPK signalling pathway is another of the main downstream effectors of RAS signalling. We wondered if its inhibition with the MEK inhibitor trametinib would have an effect in cell migration. Pik3ca^WT^ cells treated with trametinib showed a decrease in EGF-induced migration but were still able to migrate (Fig. 1e). Migration of Pik3ca^RBD^ cells was not affected by MEK inhibition. All together these results suggest that PI3K is the main RAS-effector pathway controlling cell migration and that p110α is the main PI3-Kinase isoform involved in the regulation of cell migration, although it is likely that other mechanisms may also play a role.

Next we measured the ability of EGF to activate RAS in both wild type and RBD-mutant cells. Our results showed that although basal RAS activity is lower in the RBD-mutant cells, RAS is activated normally in response to EGF in both Pik3ca^RBD^ and Pik3ca^WT^ MEFs (Fig. 1g), indicating that the migration defect in Pik3ca^RBD^ cells is not due to a defect in RAS activation.

We have previously shown that Pik3ca^RBD^ MEFs have a defect in Akt activation in response to EGF and HGF, but not in response to PDGF or FBS (ref. 16). To investigate whether the RAS-PI3-Kinase interaction could be regulating cell motility via Akt signalling, we next determined whether Pik3ca^RBD^ cells expressing inducibly activatable Akt (ER-myrAkt) could migrate in response to EGF stimulation. Our results show that restoring Akt signal in the Pik3ca^RBD^ cells also restore the defect in migration (Fig. 1h). It was noticeable that migration was partially restored even before adding 4-hydroxytamoxifen (4-HT) to the cells. Thus, we checked if the construct was leaky by analysing Akt phosphorylation and one of its targets, S6 phosphorylation. Although we were not able to detect any Akt activity in the cells prior 4-HT addition, we observed that there was an increase in p-S6 in mutant cells containing the ER-myrAkt construct in the absence of 4-HT prior EGF stimulation (Supplementary Fig. 1f), suggesting that the construct might have some basal activity.

### RAS-PI3-Kinase pathway regulates cell polarity and invasion

**Figure 2:**
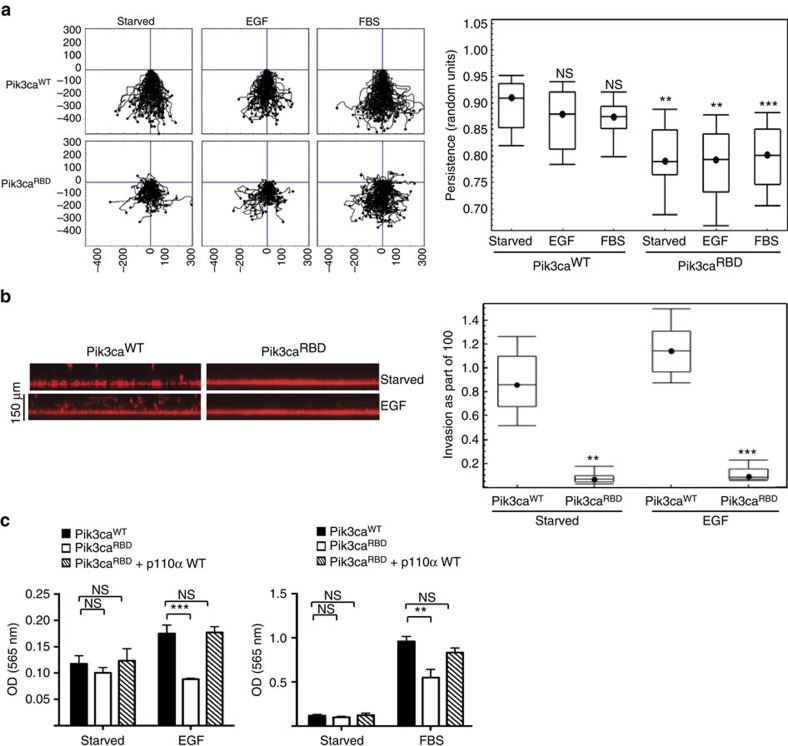
Disruption of RAS interaction with PI3-Kinase disturbs cell polarity and invasion. (**a**) Wounded Pik3ca^WT^ and Pik3ca^RBD^ MEFs monolayers were allowed to migrate for 18 h in the presence or absence EGF (20 ng ml^−1^) or FBS (10%). Migration was analysed by time-lapse video microscopy. For each condition 90 cells were tracked and persistence in the directionality of migration was analysed using Mathematica software. Analysis of variance (ANOVA) statistical analysis was performed with starved cells used as reference for each condition (NS, not significant; **P<0.05; ***P<0.005). (**b**) Invasion of Pik3ca^WT^ and Pik3ca^RBD^ cells in a collagen I matrix in the presence or absence of EGF (0.5 μg ml^−1^). Stacks are acquired from the bottom of the well over 150 μm upward. Invasion through the collagen layer was monitored in a confocal microscope and analysed using Mathematica software. ANOVA statistical analysis was performed (**P<0.05; ***P<0.005). (**c**) Invasion of Pik3ca^WT^, Pik3ca^RBD^ and Pik3ca^RBD^ WT p110α MEFs in transwells containing a layer of matrigel (growth factor reduced matrigel). Invasion was measured in either 0.2% FBS (starved), EGF (50 ng ml^−1^) or FBS (10%). Invasive cells (on the lower part of the transwell, attached to the membrane) were stained with crystal violet and then lysed using acetic acid. Assays were carried out in triplicate, with error bars indicating s.d. The results of two different experiments are shown. Error bars indicate s.d. (Significance using Student's *t*-test. NS, not significant; **P<0.05; ***P<0.005).

Migration is a polarized cellular process in which cells display a protrusive front edge that opposes a retracting trailing edge. Without this polarity, cells would lack the ability to move in a specific direction^3^,^17^,^18^. To elucidate whether the RAS-PI3-Kinase interaction has a role in establishing cell polarity, we performed wound-healing assays to measure persistence of migration direction in response to EGF and FBS stimulation. We observed that Pik3ca^RBD^ cells migrate in a disorderedly manner, whereas Pik3ca^WT^ cells migrate with a greater directional persistence towards the wound area (Fig. 2a).

Directed cell migration requires cell polarization and adhesion turnover, in which the actin cytoskeleton and microtubules play critical roles. Microtubule network polarity and stabilization controls the establishment and maintenance of the spatial and temporal coordination of migration events and is therefore the key to persistent directed migration^19^,^20^,^21^. We investigated whether the lack of directional movement of RBD-mutant cells was due to a defect in the regulation of microtubule stability. Protein levels of α-tubulin measured by western blot and immunofluorescence were similar in Pik3ca^WT^ and Pik3ca^RBD^ cells (Supplementary Fig. 2a,b). Protein levels of glutamylated tubulin, one of the major modifications to stabilize microtubules, were also similar in both cell lines. However we found that Pik3ca^RBD^ cells had an increase in the acetylation of α-tubulin, the other main microtubule stabilization process (Supplementary Fig. 2a). This difference was neither dependent nor affected by EGF treatment or other growth factors (Supplementary Fig. 2c). Immunofluorescence studies also revealed differences in the localization of acetylated α-tubulin, which in the Pik3ca^RBD^ cells is not found in the plasma membrane but rather in the perinuclear region (Supplementary Fig. 2b). We were able to partially reverse the increase in α-tubulin acetylation of mutant cells when we re-expressed a wild-type version of 110α in these cells (Supplementary Fig. 2d), thereby confirming that this difference is a consequence of disrupting the RAS-PI3-Kinase interaction.

Invasive migration is a fundamental aspect of cellular processes such as angiogenesis, embryonic development, immune response, metastasis and invasion of cancer cells. We investigated whether interaction between RAS and PI3-Kinase is required for the ability of cells to invade through a collagen matrix towards a growth factor using a previously described assay^22^. We found that although Pik3ca^WT^ cells were able to invade collagen towards EGF, Pik3ca^RBD^ cells stayed mostly in the bottom of the plate, indicating a deficiency in their invasive ability (Fig. 2b). Similar results were obtained in transwell assays in which cells have to invade through a matrigel layer. The invasive defect displayed by Pik3ca^RBD^ cells in this assay could be rescued by expression of a WT p110α (Fig. 2c).

### Rac-GTPase activity is impaired in Pik3ca^RBD^ cells

**Figure 3:**
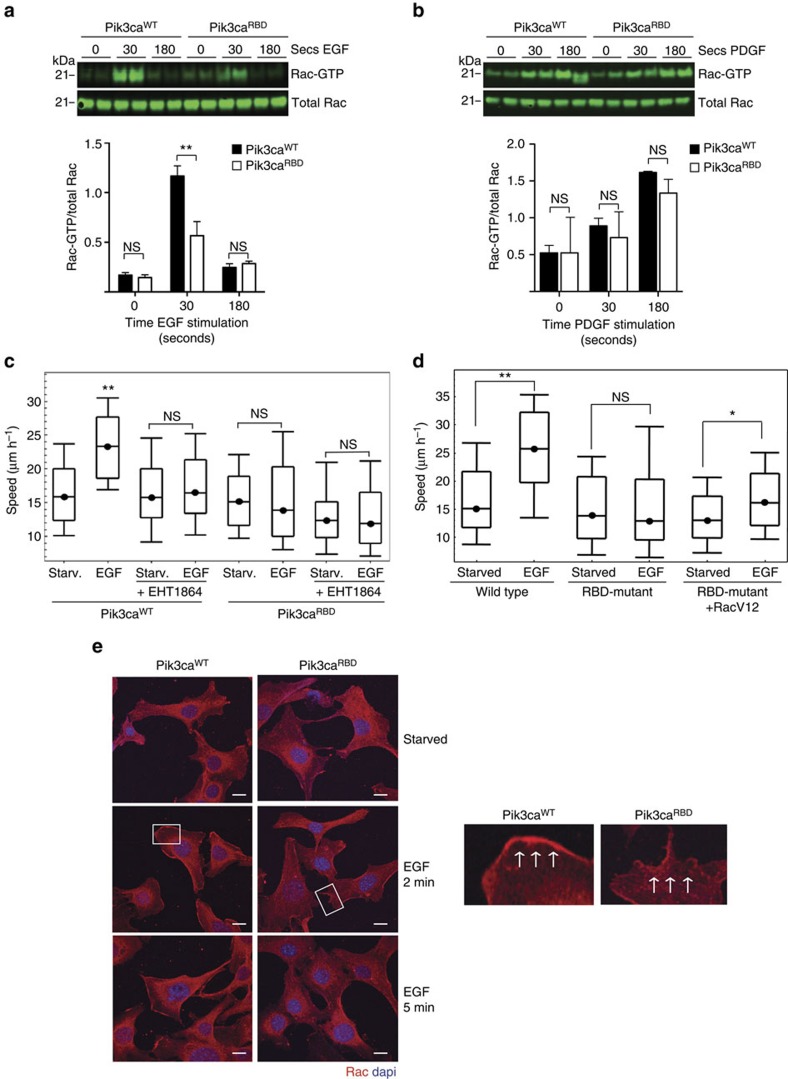
Defects in Rac-GTPase activation in Pik3ca^RBD^ cells. (**a**) MEFs were stimulated with EGF (20 ng ml^−1^) for the indicated time periods. Rac-GTP activity was established in pull-down assays using GST-PBD of PAK1 (GST-PBD). Both total lysates and proteins bound to GST-PBD were analysed by western blot to detect Rac. (**b**) MEFs were stimulated with PDGF (20 ng ml^−1^) for the indicated time periods and pull-down assays and analysis of the results were done in the same way as described for previous panel. (**c**) EGF-induced random migration of Pik3ca^WT^ and Pik3ca^RBD^ cells was analysed by time-lapse video microscopy and cell tracing in the presence or absence of the Rac inhibitor EHT-1864 (5 μM). Analysis of variance (ANOVA) statistical analysis was performed with starved cells used as reference for each condition (NS, not significant; **P<0.01). Box and whisker plot was generated as indicated for Fig. 1a. (**d**) EGF-induced random migration of Pik3ca^WT^, Pik3ca^RBD^ and Pik3ca^RBD^+RacV12 cells was analysed by time-lapse video microscopy and cell tracing. ANOVA statistical analysis was performed with starved cells used as reference for each condition (NS, not significant; **P<0.01). Box and whisker plot was generated as indicated for Fig. 1a. (**e**) Pik3ca^WT^ and Pik3ca^RBD^ cells were stimulated with EGF for the denoted time points. immunofluorescence (IF) to detect Rac accumulation in the plasma membrane was performed. DAPI co-staining was carried out to distinguish individual cells. Scale bar, 10 μm. White squares indicate part of the membrane magnified in the right hand images. Arrows indicate direction of cell movement.

Cell migration requires that Rac GTPases promote formation of actin polymers at the cell leading edge^18^,^23^,^24^. We investigated whether disruption of RAS signalling through PI3-Kinase had an impact on Rac activity and found a decrease in Rac-GTP levels in Pik3ca^RBD^ cells in response to EGF (Fig. 3a). We obtained similar results with an independent pair of MEF preparations, indicating that this difference is not due to a clonal or immortalization artefact (Supplementary Fig. 3a). Interestingly, no differences in Rac-GTP levels were found in response to stimulation with PDGF (Fig. 3b).

We then investigated whether the impairment in Rac activity was causing the migration defect observed in the Pik3ca^RBD^ cells and found that the EGF-dependent induction of migration of Pik3ca^WT^ fibroblasts was abrogated by treatment with the Rac inhibitor EHT-1864 (Fig. 3c). This did not have a significant effect on the slower migration of Pik3ca^RBD^ cells. Furthermore, the migration defect in Pik3ca^RBD^ cells was restored after expression of an active Rac construct (RacV12; Fig. 3d). Additionally, we observed a defect in Rac accumulation at the leading edge of RBD-mutant fibroblasts after EGF stimulation (Fig. 3e). These results suggest that RAS signalling through PI3-Kinase is required to mobilize Rac at the leading edge of migrating cells during EGF-induced cell motility, a key event for the establishment of cell polarity during migration^3^,^17^,^18^.

We also looked for differences in the actin cytoskeleton after disruption of the RAS-PI3-Kinase signalling. However, we did not find significant differences between Pik3ca^WT^ and Pik3ca^RBD^ cells (Supplementary Fig. 3b).

Taken together, our results suggest that RAS signalling through PI3-Kinase regulates EGF-induced cell motility by activating Rac and keeping it at the leading edge of migrating cells.

### RAS-PI3-Kinase interaction regulates Reelin expression

**Figure 4:**
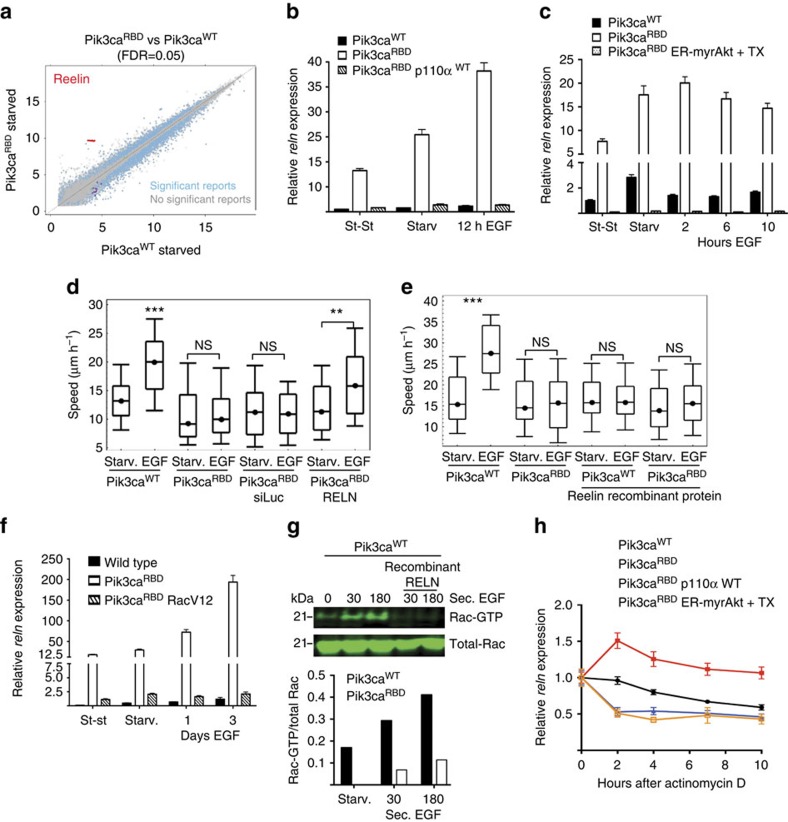
Reelin expression is regulated by RAS-PI3-Kinase pathway and is involved in migration. (**a**) Graphical display of statistical analysis performed to identify genes undergoing significant changes of expression in Pik3ca^RBD^ cells as compared with wild-type counterparts. Statistically significant probes are shown in light blue (0.05 fdr). Reln reports are shown in red. (**b**) RNA from steady-state (st-st), serum-starved or EGF-treated (20 ng ml^−1^) fibroblasts was obtained and Reln mRNA levels measured by quantitative PCR (qPCR). Actin expression was used as an internal control for normalization. Independent triplicates were used for each time point. Error bars indicate mean±s.e.m. (**c**) Levels of Reln expression were measured by qPCR in Pik3ca^RBD^ cells containing an inducible active Akt construct (ER-MyrAKT) in the presence or absence of 4-hydroxytamoxifen (TX; 100 nM). Actin expression was used as an internal control for normalization. Independent triplicates were used for each time point. Error bars indicate mean±s.e.m. (**d**) Random migration after Reln silencing in Pik3ca^RBD^ cells. Migration was analysed by time-lapse video microscopy and cell tracing in the presence or absence of EGF (20 ng ml^−1^). Box and whisker plot was generated as indicated for Fig. 1a.ANOVA statistical analysis was performed with starved cells used as reference for each condition (NS, not significant; **P<0.01; ***P<0.001). (**e**) Recombinant Reln (1 μg ml^−1^) was added to the media of Pik3ca^WT^ and Pik3ca^RBD^ cells and random migration was then analysed in the same way as described for previous panel. (**f**) *Reln* expression levels in Pik3ca^WT^, Pik3ca^RBD^ and Pik3ca^RBD^ RacV12 cells. Actin expression was used as an internal control for normalization. Independent triplicates were used for each time point. (**g**) Rac pull-down analysis in the presence of recombinant Reln. Recombinant Reln was added to the media of Pik3ca^WT^ MEFs and then Rac-GTP activity was determined in pull-down assays using GST-PBD of PAK1 (GST-PBD). Both total lysates and proteins bound to GST-PBD were analysed by western blot to detect Rac. (**h**) Representative graph showing Reln mRNA half-life. Pik3ca^WT^, Pik3ca^RBD^, Pik3ca^RBD^ p110a WT and Pik3ca^RBD^ ER-myrAkt (+TX) cells were treated with actinomycin D and levels of Reln mRNA were determined by qPCR at the displayed time points. Actin expression was used as an internal control for normalization. Independent triplicates were used for each time point.

To find regulators of migration whose expression may be controlled by RAS-PI3-Kinase signalling we performed expression analysis of non-stimulated Pik3ca^RBD^ and Pik3ca^WT^ mouse embryo fibroblasts using commercial oligonucleotide microarrays. The plot in Fig. 4a highlights gene probe sets that are differentially expressed (FDR=0.05) between Pik3ca^WT^ and Pik3ca^RBD^ cells. Supplementary Table 1 lists the top 50 probe sets that were upregulated, and Supplementary Table 2 the top 50 probe sets that were downregulated, in the Pik3ca^RBD^ cells when compared with their WT counterparts. We observed that Reelin (*RELN*) expression was highly upregulated in the Pik3ca^RBD^ cells, with nine of the top 10 upregulated reporters being Reelin sequences. We used real-time quantitative PCR to confirm that *Reelin* expression was upregulated in the Pik3ca^RBD^ cells (Fig. 4b). Importantly, *RELN* expression levels were reduced to normal levels by expression of WT p110α (Fig. 4b) and also when Akt signalling was restored in the Pik3ca^RBD^ fibroblasts (Fig. 4c). About 80% of the mutations in the coding sequence of PIK3CA map to three hot spots. Two of the hot spots, represented by the single amino acid substitutions E542K and E545K, are localized in the helical domain of the protein, the third, represented by the H1047R substitution, resides in the kinase domain^25^. We hypothesized that active PIK3CA would downregulate *RELN* expression in our Pik3ca^RBD^ cells. Stable expression of PIK3CA^H1047R^ in Pik3ca^RBD^ cells significantly decreased Reelin expression to levels compared with Pik3ca^WT^ cells (Supplementary Fig. 4a). Additionally, transwell assays showed that expression of PIK3CA^H1047R^ in Pik3ca^RBD^ cells rescued the defect in migration (Supplementary Fig. 4b). All together, our results suggested that p110α regulates expression of Reelin.

Reelin is a secreted extracellular matrix glycoprotein that helps regulate processes of neuronal migration and positioning in the developing brain by controlling cell–cell interactions. In the adult brain it modulates synaptic plasticity by enhancing the induction and maintenance of long-term potentiation. It also regulates the continuing migration of neuroblasts generated in adult neurogenesis sites^26^,^27^,^28^. Reelin expression is lost in some cancers such as breast or pancreatic cancers and is associated with a poor prognosis^29^,^30^.

We investigated whether the upregulation of *RELN* expression played a role in the migration defect observed in the Pik3ca^RBD^ cells and found that knocking down *RELN* expression with short interfering RNA restored the EGF-induced increase in migration in Pik3ca^RBD^ cells (Fig. 4d and Supplementary Fig. 4c). Moreover, addition of purified recombinant Reelin to the medium of Pik3ca^WT^ cells abrogated the increase in migration induced by EGF (Fig. 4e) but not by PDGF (Supplementary Fig. 4d).

We also found that *RELN* expression was notably decreased after expression of a constitutively activated Rac (RacV12) in Pik3ca^RBD^ cells (Fig. 4f) and that treatment with the Rac inhibitor EHT-1864 increased the expression of *RELN* in the Pik3ca^WT^ cells (Supplementary Fig. 4e). Interestingly, Rac activation by EGF in Pik3ca^WT^ cells was abrogated by the addition of purified recombinant Reelin to the medium (Fig. 4g). This suggests that there is a reciprocal regulation between Rac and Reelin, with mutual negative feedback loops. The defect in EGF-induced migration in PI3-Kinase RBD mutant cells is due to changes in expression of *RELN* and impairment of Rac activity.

We then aimed to investigate if activation of Rac or Reelin pathway were involved in the increased acetylation of microtubules. Knockdown of *RELN* in Pik3ca^RBD^ cells did not restore levels of acetylated α-tubulin (Supplementary Fig. 4f). However expression of constitutively active Rac in Pik3ca^RBD^ cells decreased the levels of acetylated tubulin in Pik3ca^RBD^ cells to levels comparable with Pik3ca^WT^ cells (Supplementary Fig. 4g). These data suggest that the increased acetylation of microtubules is dependent on Rac signalling but the mechanism by which this regulation happens is independent of Reelin pathway.

It has been described that the PI3-Kinase pathway regulates the activity of RNA-binding factors that stabilize mRNA, thus controlling gene expression^31^,^32^,^33^. The 3′-UTR region of the *RELN* gene has AU- and U-rich sequences (Supplementary Fig. 4h), suggesting that its expression might be regulated through mRNA stabilization. Indeed, disrupting the interaction between RAS and PI3-Kinase leads to an increase in the stability of *RELN* mRNA in the Pik3ca^RBD^ cells that can be reverted by expression of WT p110α or AKT (Fig. 4h).

### Reelin upregulates E-cadherin in Pik3ca^RBD^ cells

**Figure 5:**
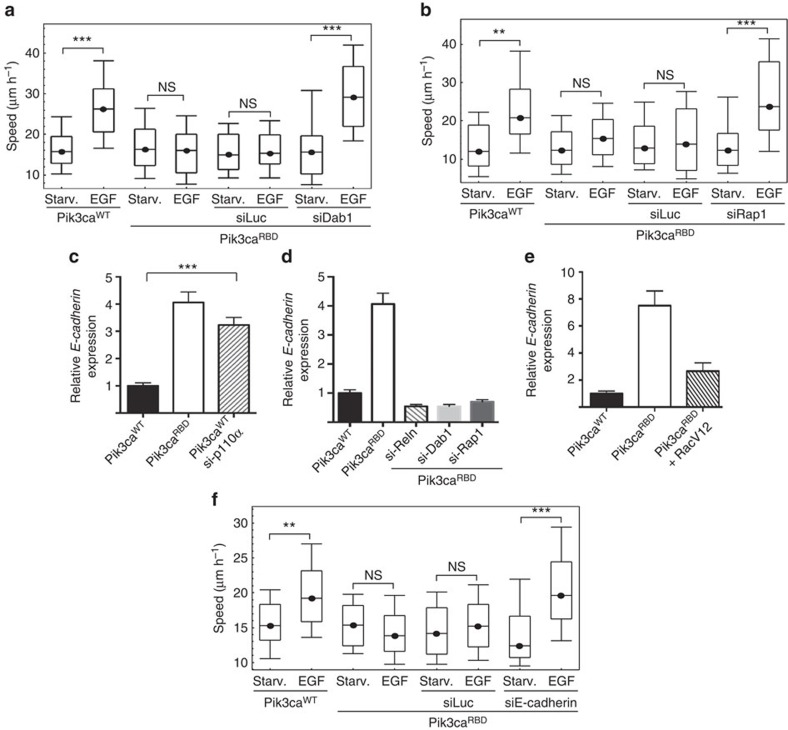
The Reelin pathway regulates migration in Pik3ca^RBD^ cells. (**a**) Random migration after *dab1* silencing in Pik3ca^RBD^ cells. Migration was analysed by time-lapse video microscopy and cell tracing in the presence or absence of EGF (20 ng ml^−1^). Box and whisker plot was generated as indicated for Fig. 1a. Analysis of variance (ANOVA) statistical analysis was performed with starved cells used as reference for each condition (NS, not significant; ***P<0.001). (**b**) Random migration after *Rap1* silencing in Pik3ca^RBD^ cells. Migration assay was performed and analysed as described for previous panel. ANOVA statistical analysis was performed with starved cells used as reference for each condition (NS, not significant; **P<0.01; ***P<0.001). (**c**) Representative graph showing *Cdh1* expression levels in Pik3ca^WT^, Pik3ca^RBD^ and Pik3ca^WT^ cells 72 h after silencing of p110α. Actin expression was used as an internal control for normalization. The assay was performed three times and each time it was performed in triplicates. Error bars indicate mean±s.e.m. (Significance using Student's *t*-test ***P<0.001). (**d**) Representative graph showing *Cdh1* expression levels in Pik3ca^RBD^ cells 72 h after silencing of *Reln*, *Dab1* or *Rap1*. Actin expression was used as an internal control for normalization. The assay was performed three times and each time it was performed in triplicates. Error bars indicate mean±s.e.m. (**e**) Representative graph showing *Cdh1* expression levels in Pik3ca^WT^, Pik3ca^RBD^ and Pik3ca^RBD^ RacV12 cells. Actin expression was used as an internal control for normalization. The assay was performed three times and each time it was performed in triplicates. Error bars indicate mean±s.e.m. (Significance using Student's *t*-test ***P<0.001). (**f**) Random migration after *Cdh1* silencing in Pik3ca^RBD^ cells. Migration assay was performed and analysed as described for **a**). Box and whisker plot was generated as indicated for Fig. 1a. ANOVA statistical analysis was performed with starved cells used as reference for each condition (NS, not significant; **P<0.01).

RELN binds to very low density lipoprotein receptor (VLDLR) and apolipoprotein E receptor 2 (ApoER2) (both from the low-density lipoprotein family), leading to induction of phosphorylation of the cytoplasmic adaptor protein Dab1 on tyrosine 198 (refs 34, 35, 36). Dab1 is, therefore, the initial effector of the Reelin-signalling cascade. We wondered if knockdown of Dab1 would have an effect in migration of our cells. Random migration assays showed that silencing of Dab1 reverted the migration defect of Pik3ca^RBD^ cells (Fig. 5a and Supplementary Fig. 5a).

Reelin, via Dab1 activation, activates Rap1 (ref. 37), so we checked whether silencing of Rap1 also reverted the migratory defect of Pik3ca^RBD^ cells. Random migration assays confirmed the involvement of Rap1 in RAS-PI3-Kinase induced migration (Fig. 5b and Supplementary Fig. 5b).

Rap1 controls cell adhesion by regulating two prominent classes of adhesion receptors, integrins and cadherins^38^,^39^. We found that in Pik3ca^RBD^ cells E-cadherin (*Cdh1*) levels were higher than in their wild-type counterparts and that knocking down p110α expression in the Pik3ca^WT^ cells resulted in increased E-cadherin expression (Fig. 5c). FACS analysis confirmed an increase in membrane-associated E-cadherin in Pik3ca^RBD^ cells (Supplementary Fig. 5c). Similarly, silencing of *RELN*, *Dab1* or *Rap1* in Pik3ca^RBD^ and Pik3ca^WT^ cells decreased *Cdh1* levels (Fig. 5d and Supplementary Fig. 5d). *Cdh1* levels were rescued by restoring Rac activation in Pik3ca^RBD^ cells (Fig. 5e). Additionally, knockdown of *Cdh1* also increased migration in response to EGF in Pik3ca^RBD^ cells (Fig. 5f and Supplementary Fig. 5e).

### Pik3ca^RBD^-derived lung tumours have increased Reln and Cdh1

**Figure 6:**
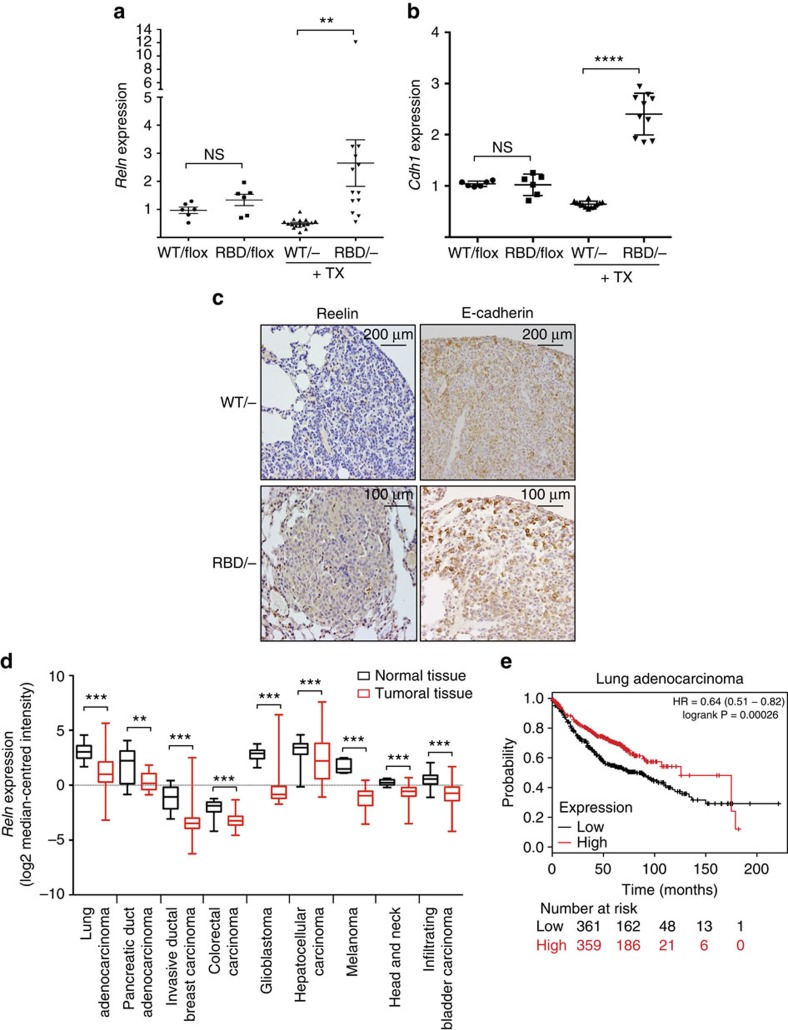
Reelin expression in tumours in mice and clinical data sets. (**a**) *Reln* expression in lung tumours from 7-week-old Pik3ca^WT/flox^, Pik3ca^RBD/flox^, Pik3ca^WT/−^ Pik3ca^RBD/−^ mice treated and untreated with tamoxifen. All these mice harbour an oncogenic mutation in Kras so they develop lung tumours. Tumours were collected 1 week after the end of tamoxifen treatment. Actin expression was used as an internal control for normalization. (**b**) *E-cadherin* expression in lung tumours from 7-week-old Pik3ca^WT/flox^, Pik3ca^RBD/flox^, Pik3ca^WT/−^ Pik3ca^RBD/−^ mice treated and untreated with tamoxifen. Tumours were collected 1 week after the end of tamoxifen treatment. Actin expression was used as an internal control for normalization. (**c**) Representative images of Reln and E-cadherin staining of lung sections from Pik3ca^WT/−^ Pik3ca^RBD/−^ mice. (**d**) Plot representing human RELN expression values from patients with the tumour types indicated in the figure. (**e**) Kaplan–Meier graph showing overall survival for lung adenocarcinoma patients with low and high expression of RELN. High and low RELN expression was divided by median.

We have shown that the RELN pathway is regulated by RAS-PI3-Kinase interaction in mouse embryo fibroblasts. Is this regulation also occurring *in vivo* and could it contribute to the regulation of migration in tumoral cells? To investigate this hypothesis we took advantage of an inducible mouse model developed in our lab in which one of the p110α alleles can be conditionally deleted and the other p110α allele has point mutations in the RBD (Pik3ca^RBD/flox^). These mice also have a conditional Cre recombinase (Cre-ERT2) allele targeted to the ubiquitously expressed Rosa26 locus. These mice were bred with Kras^LA2^ mice^13^, which have a mutated copy of KRAS, so that they spontaneously developed lung adenocarcinomas. By feeding them with tamoxifen we can remove the floxed allele and Pik3ca^WT/−^ or Pik3ca^RBD/−^ are expressed. Pik3ca^RBD/−^ mice present fewer and smaller tumours than their Pik3ca^WT/−^ counterparts and the incidence of higher grade tumours was also reduced^40^ according to standard histopathological characteristics^41^. We fed 4-week-old mice with tamoxifen for 2 weeks, and 1 week later lung tumours were collected from Pik3ca^WT/−^ or Pik3ca^RBD/−^ animals and RNA was extracted. We observed that after disruption of the RAS-PI3-Kinase interaction, Reelin expression in tumours from Pik3ca^RBD/−^ mice was some fivefold higher than in Pik3ca^WT/−^ mice (Fig. 6a). Similarly, we found an increase in E-cadherin expression in Pik3ca^RBD/−^ mice after tamoxifen treatment (Fig. 6b). We then determined whether *RELN* was also expressed in healthy lungs. We detected *RELN* expression in both Pik3ca^WT/−^ and Pik3ca^RBD/−^ mice lungs, although expression in Pik3ca^RBD/−^ mice lungs was higher than in WT as observed in our MEFs and in tumours (Supplementary Fig. 6a). Immunohistochemistry studies also showed an increase in RELN and E-cadherin in lung tumour sections from Pik3ca^RBD/−^ mice when compared with Pik3ca^WT/−^ mice (Fig. 6c). At this time point, tumours are partially regressing^40^.

In clinical data sets, we evaluated expression of the human Reelin gene (*RELN*) in various cancer cell types using microarray data from Oncomine to assess whether RELN expression is reduced in cancers. We looked for data sets in which Reelin expression was downregulated in tumours compared with healthy tissue with a *P* value below 0.05. Several studies matched these criteria (see list in ‘Methods' section), confirming that in several tumour types RELN expression is statistically significantly decreased when compared with their normal tissue counterparts (Fig. 6d).

Furthermore, we analysed the association between *RELN* expression and patients' overall survival using the KM-plotter database (http://kmplot.com)^42^. This database utilizes gene expression data and relapse free, and overall survival information from Gene Expression Omnibus (GEO) (Affymetrix microarrays only), European Genome-phenome Archive (EGA) and The Cancer Genome Atlas (TCGA), integrating gene expression and clinical data. We selected data from lung adenocarcinoma patients (*n*=720) and split samples between high and low expression of *RELN* by median, and explored whether high expression of *RELN* conferred an overall survival advantage. As shown in Fig. 6e, low expression was significantly associated with patients' poor survival, indicating that loss of *RELN* expression had a negative influence on clinical outcomes. Furthermore, when we explored *RELN* expression levels in patients with stage I disease, we observed an even stronger survival advantage of patients with higher *RELN* expression (Supplementary Fig. 6b). The hazard ratio between high and low *RELN* expression was 0.64 for all lung adenocarcinoma, and 0.31 for stage I disease. However, for patients with stage II or higher disease, *RELN* expression did not significantly correlate with outcome (hazard ratio 0.92 or higher).

We also examined survival advantage in breast cancer patients (*n*=3554) and found that high expression of RELN was associated with a better relapse-free survival (Supplementary Fig. 6c). Again, this advantage was even higher for patients with grade 1 disease histology (Supplementary Fig. 6d), with hazard ratio between high and low RELN expression of 0.71 for all breast carcinoma and 0.53 for grade 1 disease. The discrimination based on RELN expression was lost in grade 2 and higher disease (hazard ratio 0.85 or higher). These data suggest that high expression of RELN at early stages of disease is a good prognostic factor. However at later stages of disease other factors or additional mutations in the tumours could overtake the effect of RELN making this pathway less relevant.

## Discussion

Metastasis remains the major driver of mortality in patients with cancer, despite decades of therapeutic development and testing. Limiting tumour invasion and metastasis through novel cancer therapeutics could slow their dissemination leading to improved clinical outcomes. A good understanding of the molecular processes governing cell migration and invasion might lead to the development of new anti-metastatic therapies that could provide additional reductions in patient morbidity and mortality.

In this study, we have attempted to understand better the role that RAS signalling through PI3-Kinase has in cell motility. We generated immortalized mouse embryo fibroblasts from a mouse model harbouring mutations in the *Pi3kca* gene encoding the catalytic p110α isoform that block its interaction with RAS (ref. 16) and found that preventing RAS/p110α binding abrogates cell migration in response to EGF, HGF, insulin or oncogenic RAS signalling.

Disruption of RAS binding to PI3-kinase impairs Rac activation in response to EGF stimulation but not PDGF, corresponding with the behaviour observed in random migration assays, suggesting that loss of Rac activation is involved in the decrease of migration observed in the RBD-mutant cells after EGF treatment. It has been shown that PI3-Kinase is involved in tumour cell motility and invasion mainly through the regulation of Rho GTPases^43^,^44^ and that RAS can cause Rac activation via PI3-Kinase inducing actin cytoskeleton rearrangement. Inhibition of PI3K activity blocks RAS induction of membrane ruffling, while activated PI3K is sufficient to induce membrane ruffling, acting through Rac (ref. 45). We also found that disruption of RAS and PI3-Kinase binding greatly increased stabilization of microtubules. This has been proven to be due to defective Rac activation as expression of active Rac (RacV12) in Pik3ca^RBD^ cells decreases microtubule stabilization back to levels observed in control cells. Involvement of Rac in microtubule dynamics has been previously observed to involve regulation the microtubule-destabilizing protein stathmin^46^. Expression of RacV12 also rescued the migration defect observed after disruption of RAS-p110α binding, although not to the levels in p110α wild-type cells; this could reflect the fact the involvement of other pathways, or the possibility that the GTPase defective RacV12 mutant does not adequately mimic the role of endogenous Rac protein activated by normal EGF-induced signalling mechanisms.

Expression analysis data showed a striking increase in the expression of *RELN* after disruption of RAS binding to p110α. Reelin is an extracellular multifunctional protein controlling migration, growth, maturation, and synaptic activity in the developing and adult brain^47^,^48^,^49^. A major function of Reelin appears to be to act as a stop signal for migrating neuronal cell populations during brain development, and it is this activity that largely accounts for the abnormal reeling gait (‘reeler') neurological phenotype of Reelin defective mice. Reelin is also expressed in peripheral tissues, including the liver, intestine, kidney, adrenal glands and pancreas, suggesting an additional role for Reelin in development and possibly in structural maintenance of these organs^50^,^51^. In this regard it has been recently described that Reelin plays a fundamental role in the correct homeostasis of the intestine^52^,^53^.

The mechanisms controlling Reelin expression are poorly understood and have been mostly gathered from tumours and from post-mortem schizophrenia brains. In both cases it was found that loss of Reelin expression is the consequence of a Dnmt1-mediated hypermethylation of the corresponding promoter^30^,^54^,^55^,^56^. However, the mechanisms governing Reelin expression in a normal physiological situation may be different, or additional regulatory mechanisms may exist. Here we have shown that RAS binding to p110α regulates expression of *Reelin* by decreasing the half-life of its mRNA. The PI3-Kinase pathway is involved in the control of gene expression regulating the activity of RNA-binding factors that stabilize mRNA^31^,^32^,^33^. Additionally, it has been shown that *Reelin* expression is also controlled by the binding of the microRNA miR-128 to the 3′-UTR region of *Reelin* mRNA^57^. Future experiments are required to fully address how RAS-PI3K signalling might be controlling Reelin mRNA stabilization.

Increased Reelin expression correlates with the decrease in migration we observed in response to EGF. However, it does not seem to have any effect on migration in quiescent cells or after PDGF stimulation. We believe this effect is due to differences in the tyrosine kinase receptor activation. Whereas EGF receptor activation engages both RAS and tyrosine-phosphorylated adaptor protein/p85 regulatory subunit binding to signal to PI3K, PDGF receptor binds directly to p85 and controls PI3K in a mechanism that is independent of RAS (ref. 58). These differences in activation might account for the differences observed in migration after stimulation by different growth factors. Thus, although RAS-PI3K binding regulates the expression of *RELN*, involvement of Reelin in migration will depend on the upstream activator.

Besides the role of Reelin in normal physiological conditions, loss of Reelin expression has been linked to tumour development and metastasis formation. This loss of expression correlates with poor prognosis in cancers and increased metastatic potential^29^,^30^,^56^,^59^. We have also confirmed that lung tumours lacking the RAS-PI3K interaction present an increase in Reelin and E-cadherin expression. It is well accepted that loss of E-cadherin is associated with increased cell migration and invasion^60^,^61^. This loss appears to be a key event in the acquisition of invasive capacity, because re-expression of E-cadherin suppresses the invasion of tumour cells *in vitro*^62^. Although a metastatic model of RAS-induced lung cancer would be necessary to test the role of Reelin and E-cadherin upregulation in tumour invasion and metastasis formation, these results suggest that RAS-PI3K regulation of Reelin might be part of a mechanism by which cancer cells could gain the enhanced motility necessary to metastasize. Finally, we found that both lung adenocarcinoma and breast carcinoma patients have better survival expectancy when Reelin levels in their tumours were higher. This effect is only seen for the lowest grade tumours, suggesting that while Reelin may be able to constrain tumour spread; this ability can be overcome by other mechanisms of tumour progression.

Overall our data demonstrate a specific role for the interaction of RAS with PI3-Kinase in the regulation of cell migration. RAS-PI3-Kinase regulation of Reelin is essential for proper cell migration and failure of regulation of this pathway might result in loss of Reelin expression and increased migratory phenotype. Ways to re-express Reelin in tumours, or to suppress Rac activation in tumours lacking Reelin expression, might be explored as a therapeutic approach to reduce cancer cell migration and to improve patient outcome.

## Methods

### Tissue culture

Primary cultures of MEFS were generated in house as previously described^63^ (all clones tested negative for mycloplasma). Immortalized cultures of MEFs were generated as described by Gupta *et al*.^16^. MEFs were cultured in Dulbecco's modified Eagle's medium supplemented with FBS (10% FBS; Hyclone, Logan, UT, USA), glutamine (2 mM), penicillin (100 U ml^−1^) and streptomycin (100 μg ml^−1^). Cultures were grown in a humidified CO_2_ (10%) atmosphere at 37 °C.

### Random migration and wound-healing assays

For random migration assays, cells were seeded in 24-well plates and 24 h later they were serum-starved overnight. Fresh Dulbecco's modified Eagle's medium containing 0.2% FBS with or without the desired growth factor at the specific concentration was added (FBS 10%; EGF 20 ng ml^−1^; PDGF 20 ng ml^−1^; FGF2 10 ng ml^−1^; HGF 10 ng ml^−1^; insulin 100 ng ml^−1^).

For wound-healing assays PIK3CA^WT^ and PIK3CA^RBD^ cells were seeded at confluency in 6-well plates and subjected to scratch wounding. A wound track was introduced by scraping the cell monolayer with a yellow pipette tip (10 μl). Media was removed and wells were washed with PBS to remove unattached and dead cells. Media with or without the desired growth factor at the specific concentration was then added (FBS 10%; EGF 20 ng ml^−1^).

For both assays duplicates of each condition and genotype were prepared. Time-lapse imaging was carried out for 18 h. One image was taken every 10 min at three different positions within the same well using a Nikon microscope driven by Metamorph (Molecular Devices, Chicago, IL, USA). A total of 90 cells per condition were tracked using CellTrack. Tracks were then analysed using a previously described Mathematica notebook^64^.

### RAS and Rac-GTPase activation assays

GTP-bound RAS/Rac was assayed using a commercial kit from Millipore (Billerica, MA, USA) and following the manufacturer's protocol. For quantitative western blotting, bound primary antibodies were detected by secondary conjugates compatible with infrared detection at 700 and 800 nm, and membranes were scanned using the Odyssey Infrared Imaging System (Odyssey, LICOR). Finally GTP-activated protein levels were normalized to total protein and plotted.

### Microarrays

The Agilent Whole Mouse Genome Microarray platform (G4122F; Agilent Technology) and one-colour experimental design were used. This microarray contains 41,174 mouse probes (features) and 93 control spots, with each feature comprised of a single 60-mer oligonucleotide probe. These probes mapped to 33,978 individual mouse transcripts (sequence IDs provided by the manufacturer). Sample labelling, hybridization to microarrays, scanning and calculation of normalized expression ratios was carried out at Oxford Gene Technology microarray facility. Sample labelling and hybridization were performed according to protocols specified by the manufacturer One-Color Microarray-Based Gene Expression Analysis. Briefly, 1 μg of total RNA and Cy3-labelled CTP fluorescent dyes were used to generate fluorescent cRNA with One-Color Microarray-Based Gene Expression Analysis (Quick Amp Labelling) v5.7 (Agilent Technologies, Palo Alto, CA, USA). For hybridization, 1.65 μg of Cy3-labelled cRNA was added on microarray slide for 17 h at 65 C at 10 r.p.m. using the Hybridization Oven kit procedure provided by Agilent Technologies. After hybridization, the slides were then washed per Agilent's SSPE wash protocol, dried and scanned at 5 μm resolution with a G2505C DNA microarray scanner (Agilent Technologies). Data were then extracted from images by the Feature Extraction software version 10.5.1.1, protocol GE1_105_Jan09 (Agilent Technologies).

The expression data was log transformed and quantile normalized using Agilent's gProcessedSignal chip quantification value as a measure of expression. Control probes were removed before normalization. Genotype-dependent transcriptional changes were identified by linear model. The model was fitted across all samples and the t-statistics moderating by empirical Bayes shrinkage. Significant transcriptional changes were selected using a 0.05 FDR threshold. The analysis was carried out using the Limma package from Bioconductor 2.6 (ref. 65) using default parameters. Annotation was obtained from the mgug4122a annotation package. The RNA-seq data have been deposited with Gene Expression Omnibus, GEO accession number GSE77843.

### Invasion assays

Invasion assays were carried out as previously described^22^. Analysis of the invasion assay image data was carried out using a custom developed Mathematica (Wolfram Research) notebook, which generates aligned normalized invasion profiles for each three-dimensional image, determines an invasion value from each profile and performs an analysis of variance on the plate invasion data. In more detail each three-dimensional image is treated in the following way. The image is imported and the Otsu method threshold value is subtracted. The resultant cell intensities are summed in x and y generating a total cell intensity with z profile of the image. The collected intensity profiles are co-aligned in z using their maxima by padding individual profiles with zero valued z-positions as necessary. This alignment corrects for variation of the cell layer position within the image stacks and allows a single-invasion horizon applicable to all profiles. Selection of an appropriate z-position as the invasion horizon is by inspection of the collected z-profiles. Invasion values for each image are calculated as the proportion of total intensity located beyond the invasion horizon. The sets of experimental group data are compared with the control group data using analysis of variance.

### Western blotting

Protein samples extracted from total cell lysates using RIPA buffer were subjected to electrophoresis commercial SDS polyacrylamide gels (Novex, Life Technologies) under reducing conditions, and subsequently transferred to polyvinylidene difluoride membranes (Millipore Immobilon-P). Protein bands were detected using the ECS system (GE Healthcare) Antibodies directed against Phospho-AKT (Ser473; 4060), Phospho-S6 (2211), Pan-Akt (2920), Cdh1 (3195), actin (4968) and tubulin (2125) were purchased from Cell Signalling Technologies. Antibody against Reln was obtained from R&D. Antibody against acetylated α-tubulin (ab2460) was obtained from Abcam. Antibodies against Rac (05–389), Ras (05–516) and Glu-tubulin (AB3201) were purchased from Millipore. All antibodies were used in a 1:1,000 dilution. Full blots scans are shown at the end of Supplementary Information.

### Immunofluorescence

Cells were fixed using 4% paraformaldehyde, permeabilized with 0.1% Triton X-100 and blocked for 1 h with 3% BSA in PBS before incubation with the primary antibodies. To stain actin fibres, Alexa Fluor 488 Phalloidin (Invitrogen) was directly added to the primary antibody mixture. Alexa Fluor 488- or Alexa Fluor 594-conjugated secondary antibodies (Invitrogen) were used to detect the indicated proteins at a 1:500 dilution. Cells were counterstained with DAPI (Sigma). Images were taken using a Zeiss LSM510 confocal microscope.

### Transwell assays

Transwell invasion assays were carried out using the commercially available kit CytoSelect 24-Well Cell Invasion Assay, Basement Membrane (Cell Biolabs, San Diego, CA, USA) and following the manufacturer instructions. EGF (50 nM) was used as a chemo-attractant to encourage cell migration.

### Short interfering RNA transfection

Cells were reverse-transfected with 25 nM of Dharmacon SMARTpools in 24-well plates using Dharmafect 4 reagent (Lafayette, CO, USA) following manufacturer instructions. Efficiency of all silencing experiments was checked by quantitative PCR and silencing of >80% of the target gene was achieved (data not shown).

### Real-time quantitative PCR

Total RNA was extracted using QIAshredder and RNAeasy mini Kit from Qiagen (Qiagen, Hilden, Germany), according to manufacturer's protocol. Total RNA (1 μg) was used as template for reverse transcription reaction (High Capacity cDNA Reverse Transcription Kit, Life Technologies Ltd, Paisley, UK). Single stranded complementary DNA products were then analysed using the 7900HT Fast Real-Time PCR system (Applied Biosystems), and specific primers for Reelin (QT00093569), E-cadherin (QT00121163), Dab1 (QT00318941), Rap1 (QT02532915) and actin (QT01136772; Quantitect primer Assay, Qiagen, Hilden, Germany).

### Clinical data analysis

Microarray expression data from Oncomine database were analysed to determine if loss of RELN expression was associated with tumour development. Statistical significant data set with *P* values below 0.0001 in which RELN expression was downregulated >1.5 times were selected (we selected: the Okayama data set for lung adenocarcinoma (GSE31210); the Grutzmann data set for pancreatic ductal adenocarcinoma (E-MEXP-950); TCGA data set (http://tcga-data.nci.nih.gov/tcga/) for invasive ductal breast carcinoma, colorectal carcinoma and glioblastoma; Mas (GSE14323) and Roessler (GSE14520) data sets for hepatocellular carcinoma; Talantov dataset for melanoma (GSE3189); Ginos data set for Head and Neck carcinomas^66^; and Sanchez-Carbayo for infiltrating bladder carcinoma^67^). Expression data values for each individual study were plotted using GraphPad PRISM version 5.b.

### Survival analysis

Kaplan–Meier graphs were constructed using the KM-plotter database (http://kmplot.com/)^42^. This database contains gene expression data and relapse free and overall survival information from GEO (Affymetrix microarrays only), EGA and TCGA. The database is handled by a PostgreSQL server, which integrates gene expression and clinical data simultaneously. To analyse the prognostic value of RELN, patient samples were split into two groups according to mean expression value. The two patient cohorts were compared by a Kaplan–Meier survival plot, and the hazard ratio with 95% confidence intervals and log rank *P* value were calculated.

### Statistical analysis

Significance was determined also with GraphPad Prism 5 software using the Student's *t*-test unless stated otherwise.

## Additional information

**Accession codes:** The RNA-seq data have been deposited in the Gene Expression Omnibus database, under accession number GSE77843.

**How to cite this article:** Castellano, E. *et al*. RAS signalling through PI3-Kinase controls cell migration via modulation of Reelin expression. *Nat. Commun.* 7:11245 doi: 10.1038/ncomms11245 (2016).

## Supplementary Material

Supplementary Information Supplementary Figures 1-6

Supplementary Data 1 The top 50 probe sets from microarray expression analysis up-regulated in Pik3ca RBD MEFs compared to WT counterparts

Supplementary Data 2 The top 50 probe sets from microarray expression analysis down-regulated in Pik3caRBD MEFs compared to WT counterparts
