# Supplementary material for: RAS signalling through PI3-Kinase controls cell migration via modulation of Reelin expression
Source: Nat Commun. 2016 Apr 13;7:11245. doi: 10.1038/ncomms11245 (PMC4833863; doi:10.1038/ncomms11245)

SUPPLEMENTARY MATERIAL

CASTELLANO et al.

Figure S1

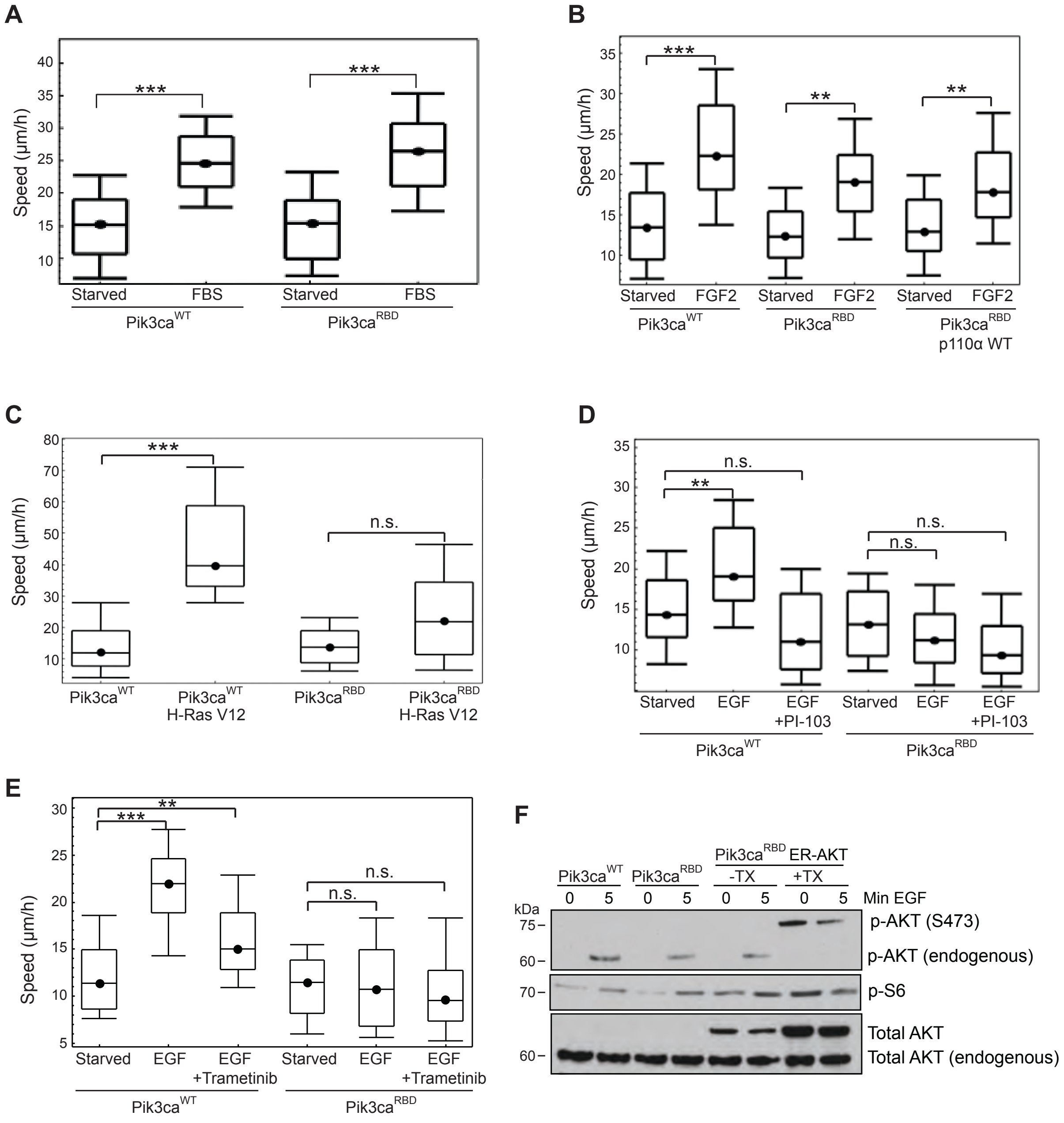

Supplementary Figure 1Disruption of RAS binding to PI3-Kinase impairs cell motility

A) Random migration of $Pik3ca^{WT}$ and $Pik3ca^{RBD}$ was analysed by time-lapse video microscopy and cell tracing in the presence or absence of FBS (10%). Cells were imaged at 10-minute intervals for 18 h. Graphs show migration tracks obtained from 90 cells in each experimental condition. The data are represented as a box and whisker plot in which the box shows the interquartile range that contains values between 25th and 75th percentile. The line inside the box show the median. The two whiskers show adjacent values. The upper adjacent value (upper mark) is the value of the largest observation that is less than or equal to the upper quartile plus 1.5 the length of the interquartile range. Analogously the lower adjacent value (lower mark) is the value of the smallest observation that is greater than or equal to the lower quartile less 1.5 times the length of interquartile range. ANOVA statistical analysis was performed with starved cells used as reference (*** $p<0.001$).

B) Random migration of $Pik3ca^{WT}$, $Pik3ca^{RBD}$ and $Pik3ca^{RBD}$ containing WT p110α was analysed by time-lapse video microscopy and cell tracing in the presence or absence of FGF (10ng/ml). Assay was carried out as described for panel A) (** $p<0.01$; *** $p<0.001$).

C) Random migration of $Pik3ca^{WT}$ H-RAS V12 and $Pik3ca^{RBD}$ H-RAS V12 cells was analysed by time-lapse video microscopy. Assay was carried out as described for panel A) (n.s. no significant; *** $p<0.001$).

D) Random migration of $Pik3ca^{WT}$ and $Pik3ca^{RBD}$ was analysed by time-lapse video microscopy and cell tracing in the presence or absence of PI-103 (100nM). Assay was carried out as described for panel A) (n.s. no significant; *** $p<0.001$).

E) Random migration of $Pik3ca^{WT}$ and $Pik3ca^{RBD}$ was analysed by time-lapse video microscopy and cell tracing in the presence or absence of trametinib (100nM). Assay was carried out as described for panel A) (n.s. no significant; (** $p<0.01$; *** $p<0.001$).

F) Western blot analysis of AKT $Pik3ca^{WT}$, $Pik3ca^{RBD}$ and $Pik3ca^{RBD}$ ER-MyrAKT cells after EGF stimulation (20 ng/ml) for the indicated time points.

Figure S2

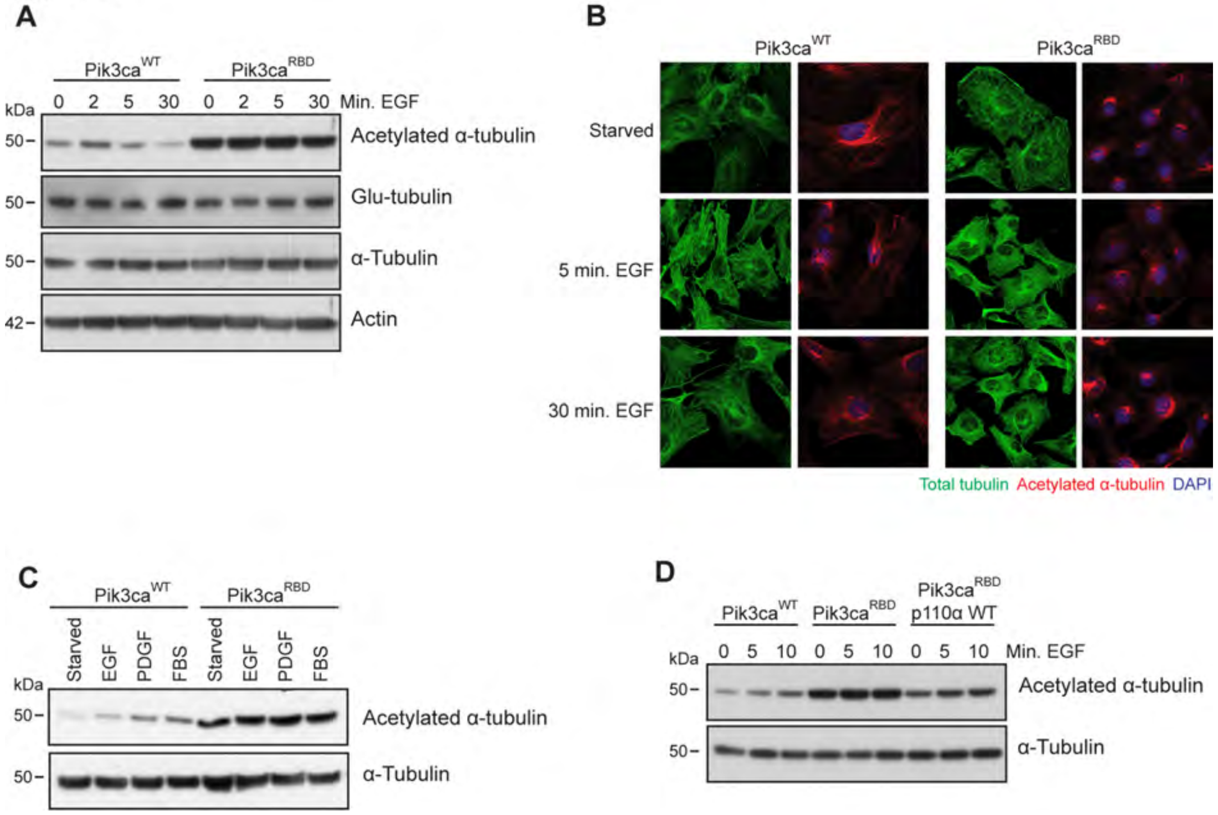

Supplementary Figure 2. RAS-PI3-Kinase interaction regulates cell polarity and invasion

A) Western blot analysis of Acetylated α-tubulin, Glu-tubulin and α-tubulin in Pik3ca^{WT} and Pik3ca^{RBD} cells after EGF stimulation (20 ng/ml) for the shown times.

B) Representative IF images of Acetylated α-tubulin (red) and α-tubulin (green) in Pik3ca^{WT} and Pik3ca^{RBD} cells.

C) Western blot analysis of Acetylated α-tubulin and α-tubulin in Pik3ca^{WT}, Pik3ca^{RBD} and Pik3ca^{RBD} p110α WT cells after EGF stimulation (20 ng/ml) for the shown times.

D) Western blot analysis of Acetylated α-tubulin in Pik3ca^{WT} and Pik3ca^{RBD} cells after stimulation with EGF (20 ng/ml), PDGF (20ng/ml) or FBS (10%) for 15 minutes.

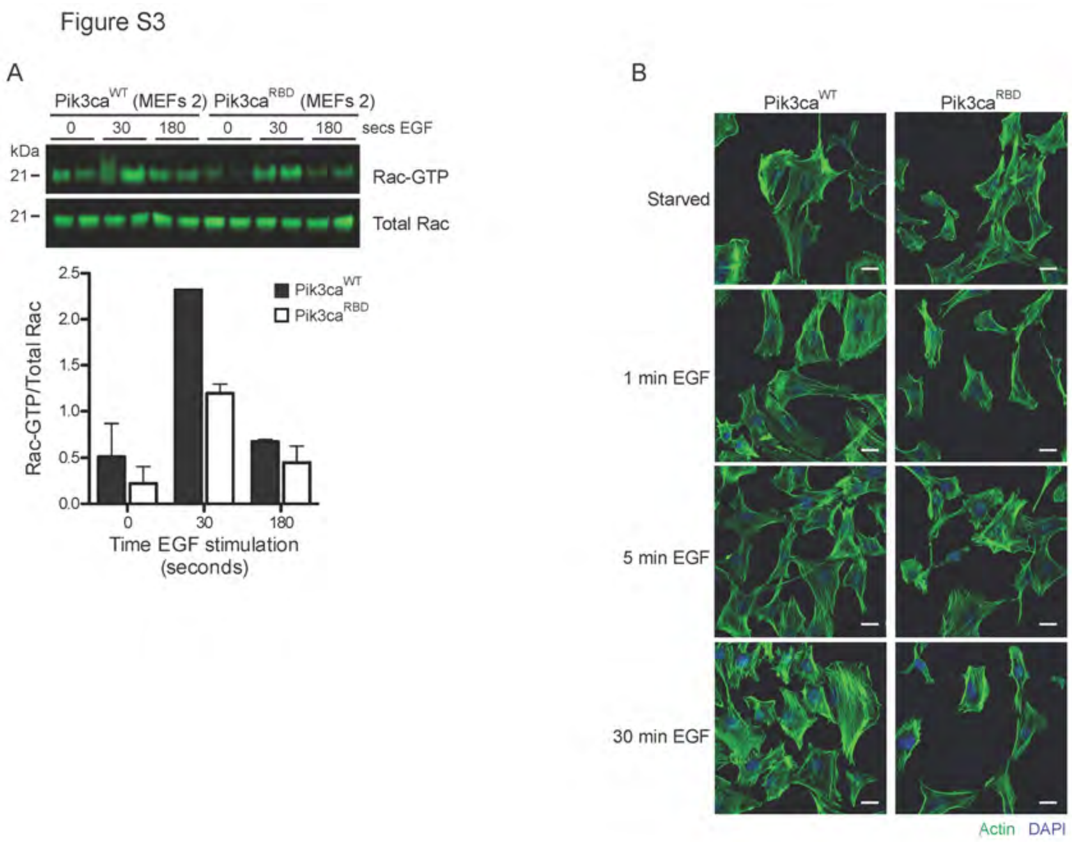

Supplementary Figure 3. Rac activity is impaired in Pik3ca^{RBD} cells

A) MEFs were stimulated with EGF (20ng/ml) for the indicated time periods. Rac-GTP activity was established in pull-down assays using GST-CRIB of PAK1 (GST-PAKcrib). Both total lysates and proteins bound to GST-PAKcrib were analyzed by western blot to detect Rac. Band intensity of the total lysates was used to normalize band intensity of Rac-GTP fraction and values were represented.

B) Representative IF images of the actin cytoskeleton in Pik3ca^{WT} and Pik3ca^{RBD} cells. Scale bar 20μm.

Figure S4

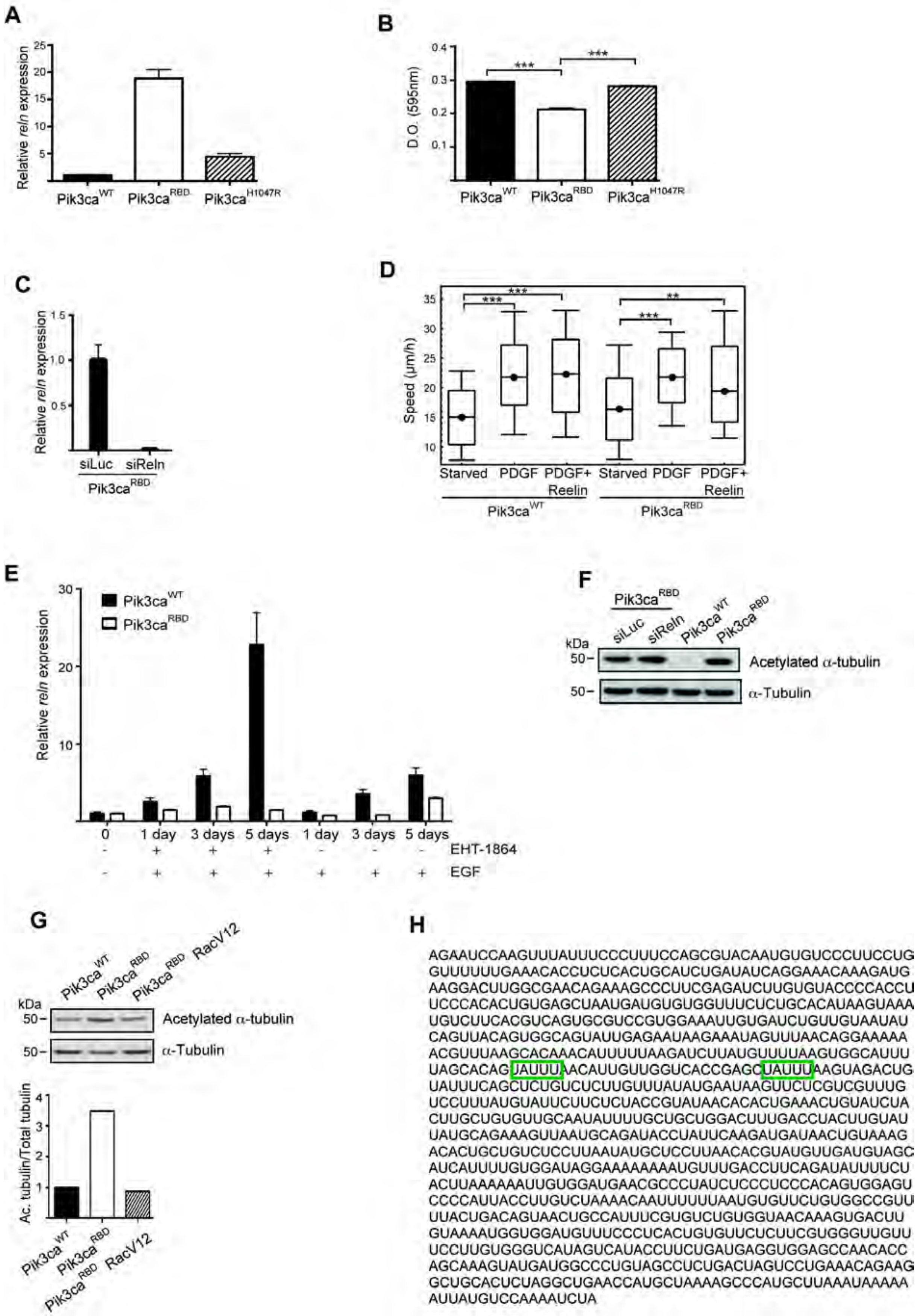
A
Relative reln expression
Pik3ca WT
Pik3ca RBD
Pik3ca H1047R
B
D.O. (595nm)
***
***
Pik3ca WT
Pik3ca RBD
Pik3ca H1047R
C
Relative reln expression
siLuc
siReln
Pik3ca RBD
D
Speed (µm/h)
***
***
***
**
Starved
PDGF
PDGF+ Reelin
Starved
PDGF
PDGF+ Reelin
Pik3ca WT
Pik3ca RBD
E
Relative reln expression
Pik3ca WT
Pik3ca RBD
0
1 day
3 days
5 days
1 day
3 days
5 days
EHT-1864
EGF
F
Pik3ca RBD
siLuc
siReln
Pik3ca WT
Pik3ca RBD
kDa
50-
50-
Acetylated α-tubulin
α-Tubulin
G
Pik3ca WT
Pik3ca RBD
Pik3ca RBD RacV12
kDa
50-
50-
Acetylated α-tubulin
α-Tubulin
Ac. tubulin/Total tubulin
Pik3ca WT
Pik3ca RBD
Pik3ca RBD RacV12
H
AGAAUCCAAGUUUAUUUCCCUUUCCAGCGUACAAUGUGUCCCUUCCUG
GUUUUUUGAAACACCUCUCACUGCAUCUGAUAUCAGGAAACAAAGAUG
AAGGACUUGGCGAACAGAAAGCCCUUCGAGAUCUUGUGUACCCCACCU
UCCCACACUGUGAGCUAAUGAUGUGUGGUUUCUCUGCACAUAAGUAAA
UGUCUUCACGUCAGUGCGUCCGUGGAAAUUGUGAUCUGUUGUAAUAU
CAGUUACAGUGGCAGUAUUGAGAAUAAGAAAUAGUUUAACAGGAAAAA
ACGUUUAAGCACAAACAUUUUUAAGAUCUUAUGUUUUAAGUGGCAUUU
UAGCACAGUAUUUAACAUUGUUGGUCACCGAGCUAUUUAAGUAGACUG
UAUUUCAGCUCUGUCUCUUGUUUAUAUGAAUAAGUUCUCGUCGUUUG
UCCUUUAUGUAUUCUUCUCUACCGUAUAACACACUGAAACUGUAUCUA
CUUGCUGUGUUGCAAUAUUUUGCUGCUGGACUUUGACCUACUUGUAU
UAUGCAGAAAGUUAAUGCAGAUACCUAUUCAAGAUGAUAACUGUAAAG
ACACUGCUGUCUCCUUAAUAUGCUCCUUAACACGUAUGUUGAUGUAGC
AUCAUUUUGUGGAUAGGAAAAAAAAUGUUUGACCUUCAGAUAUUUUCU
ACUUAAAAAAUUGUGGAUGAACGCCCUAUCUCCCUCCCACAGUGGAGU
CCCCAUUACCUUGUCUAAAACAAUUUUUUAAUGUGUUCUGUGGCCGUU
UUACUGACAGUAACUGCCAUUUCGUGUCUGUGGUAACAAAGUGACUU
GUAAAAUGGUGGAUGUUUCCCUCACUGUGUUCUCUUCGUGGGUUGUU
UCCUUGUGGGUCAUAGUCAUACCUUCUGAUGAGGUGGAGCCAACACC
AGCAAAGUAUGAUGGCCCUGUAGCCUCUGACUAGUCCUGAAACAGAAG
GCUGCACUCUAGGCUGAACCAUGCUAAAAGCCCAUGCUUAAAUAAAAA
AUUAUGUCCAAAAUCUA

Supplementary Figure 4. Reelin expression is regulated by RAS-PI3-Kinase pathway

A) Reelin expression was checked by qPCR in $Pik3ca^{WT}$, $Pik3ca^{RBD}$ cells and $Pik3ca^{RBD}$ expressing the oncogenic p110a H1047R. Actin expression was used as an internal control for normalization. Error bars indicate mean ± SEM.

B) Transwell assays in $Pik3ca^{WT}$, $Pik3ca^{RBD}$ and $Pik3ca^{RBD}$ expressing the oncogenic p110a H1047R. EGF 100ng/mL was used as chemoattractant agent in the lower chamber of the transwell. Error bars indicate mean ± SEM. T-test was used to determine significance (*** $p<0.001$)

C) Silencing efficiency of Reelin knockdown in $Pik3ca^{RBD}$ cells was checked by qPCR. Actin expression was used as an internal control for normalization. Error bars indicate mean ± SEM.

D) Random migration of $Pik3ca^{WT}$ and $Pik3ca^{RBD}$ was analysed by time-lapse video microscopy and cell tracing in the presence or absence of PDGF (20ng/ml). Cells were imaged at 10-minute intervals for 18 h. Graphs show migration tracks obtained from 90 cells in each experimental condition. Box and whisker plot was generated as indicated in figure S1A. ANOVA statistical analysis was performed with starved cells used as reference (** $p<0.01$; *** $p<0.001$).

E) *Reln* expression levels in EGF-stimulated (20ng/ml) $Pik3ca^{WT}$ and $Pik3ca^{RBD}$ after treatment with the Rac inhibitor EHT-1864 for the indicated time points. Actin expression was used as an internal control for normalization.

F) Western blot analysis of acetylated α-tubulin in $Pik3ca^{RBD}$ cells 72h after Reln silencing. Levels of acetylated α-tubulin in $Pik3ca^{WT}$ and $Pik3ca^{RBD}$ cells are also shown.

G) Western blot analysis of acetylated α-tubulin in $Pik3ca^{WT}$, $Pik3ca^{RBD}$ and $Pik3ca^{RBD}$ RacV12 MEFs and graph showing quantification of the western blot bands.

H) 3'-UTR region of the mouse *Reln* gene transcript. Green boxes denote canonical mRNA stabilization signals (AUUUA).

Figure S5

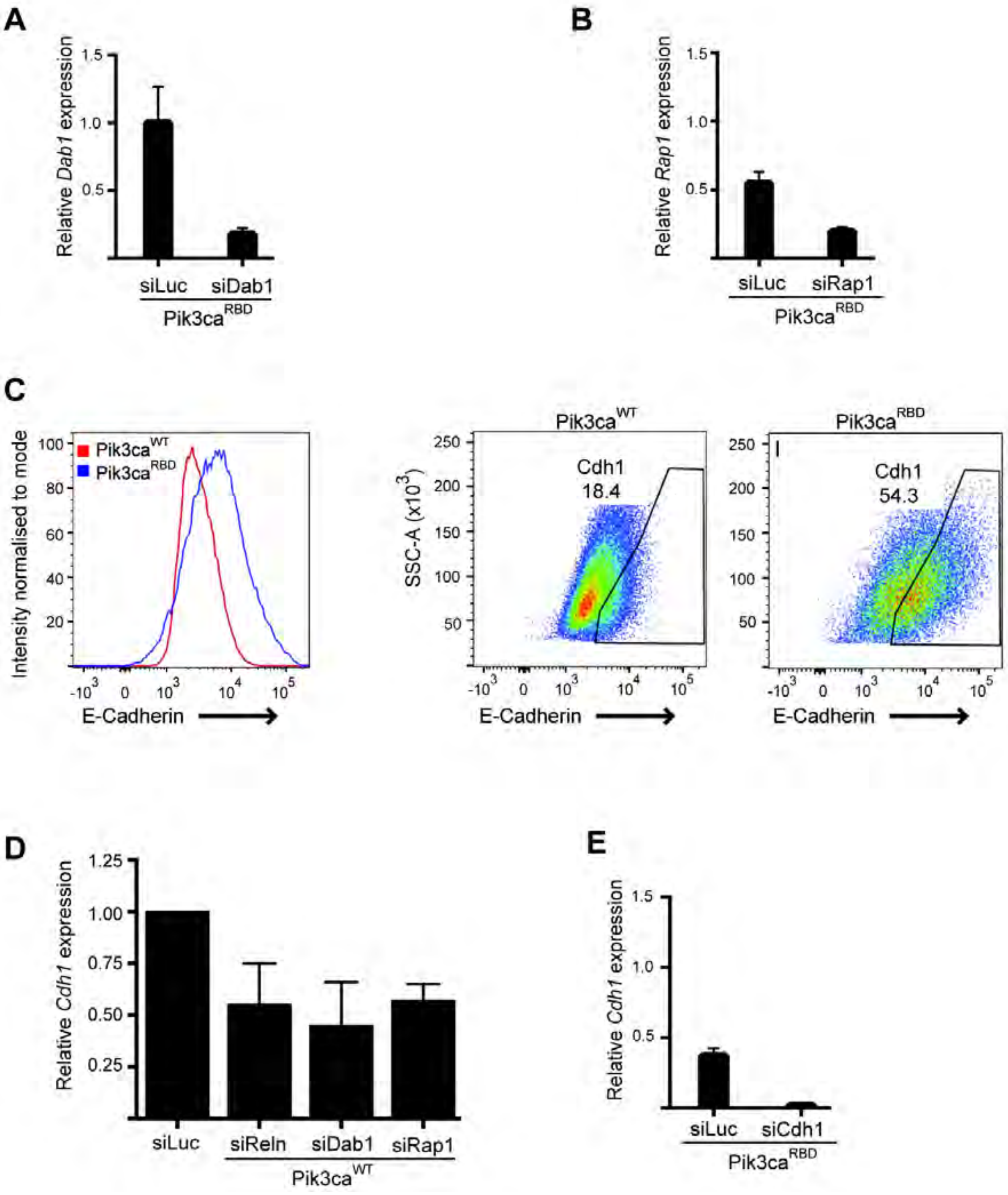
A
Relative Dab1 expression
1.5
1.0
0.5
siLuc
siDab1
Pik3ca RBD
B
Relative Rap1 expression
1.5
1.0
0.5
siLuc
siRap1
Pik3ca RBD
C
Intensity normalised to mode
100
80
60
40
20
Pik3ca WT
Pik3ca RBD
-10 3
0
10 3
10 4
10 5
E-Cadherin
Pik3ca WT
SSC-A (x10 3)
250
200
150
100
50
Cdh1
18.4
E-Cadherin
Pik3ca RBD
Cdh1
54.3
E-Cadherin
D
Relative Cdh1 expression
1.25
1.00
0.75
0.50
0.25
siLuc
siReln
siDab1
siRap1
Pik3ca WT
E
Relative Cdh1 expression
1.5
1.0
0.5
siLuc
siCdh1
Pik3ca RBD

Supplementary Figure 5. Reelin upregulates E-Cadherin in Pik3ca^{RBD} cells

A) Silencing efficiency of Dab1 knockdown in Pik3ca^{RBD} cells was checked by qPCR. Actin expression was used as an internal control for normalization. Error bars indicate mean ± SEM.

B) Silencing efficiency of Rap1 knockdown in Pik3ca^{RBD} cells was checked by qPCR. Actin expression was used as an internal control for normalization. Error bars indicate mean ± SEM.

C) FACS analysis of membrane-associated E-Cadherin in Pik3ca^{WT} and Pik3ca^{RBD} cells.

D) *Cdh1* expression levels in Pik3ca^{WT} cells 72 hours after silencing of *reln*, *dab1* or *rap1*. Actin expression was used as an internal control for normalization. Error bars indicate mean ± SEM.

E) Silencing efficiency of Cdh1 knockdown in Pik3ca^{RBD} cells was checked by qPCR. Actin expression was used as an internal control for normalization. Error bars indicate mean ± SEM.

Figure S6

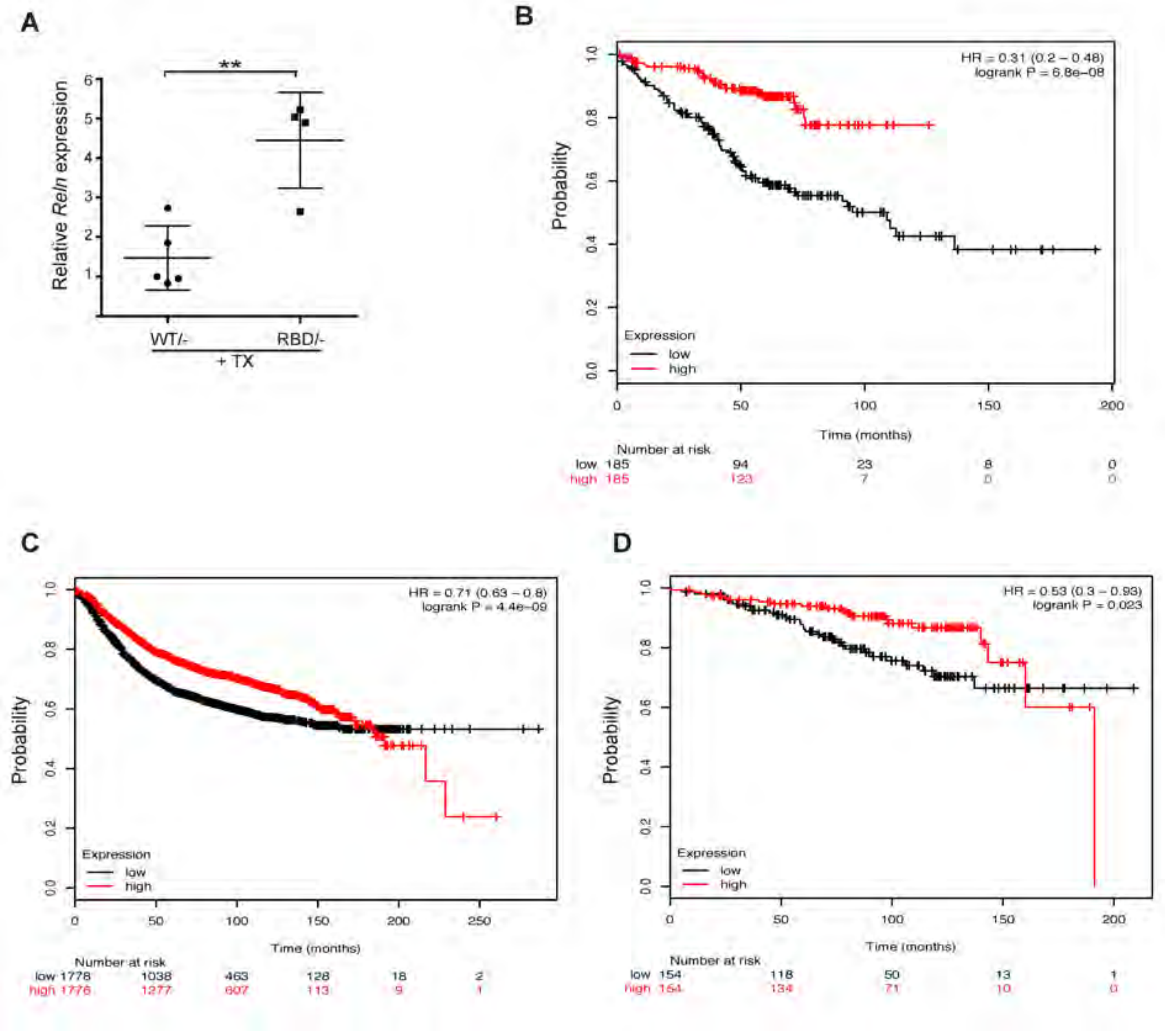

Supplementary Figure 6. Disruption of RAS binding to PI3-Kinase in lung tumours up-regulates Reelin and E-cadherin

A) *Reln* expression in healthy lungs from 7-week old Pik3ca$^{WT/-}$ Pik3ca$^{RBD/-}$ mice treated with tamoxifen. Lungs were collected one week after the end of tamoxifen treatment. Actin expression was used as an internal control for normalization.

B) Kaplan-Meier graph showing overall survival data from lung adenocarcinoma patients in stage I of disease with high or low expression of Reelin. High and low RELN expression was divided by median.

C) Kaplan-Meier graph showing the relapse free survival curve for breast cancer patients with low and high expression of RELN. High and low RELN expression was divided by median.

D) Kaplan-Meier graph showing the relapse free survival curve for breast cancer patients in grade 1 of disease with low and high expression of RELN. High and low RELN expression was divided by median.

FULL BLOTS SCANS

Figure S2A

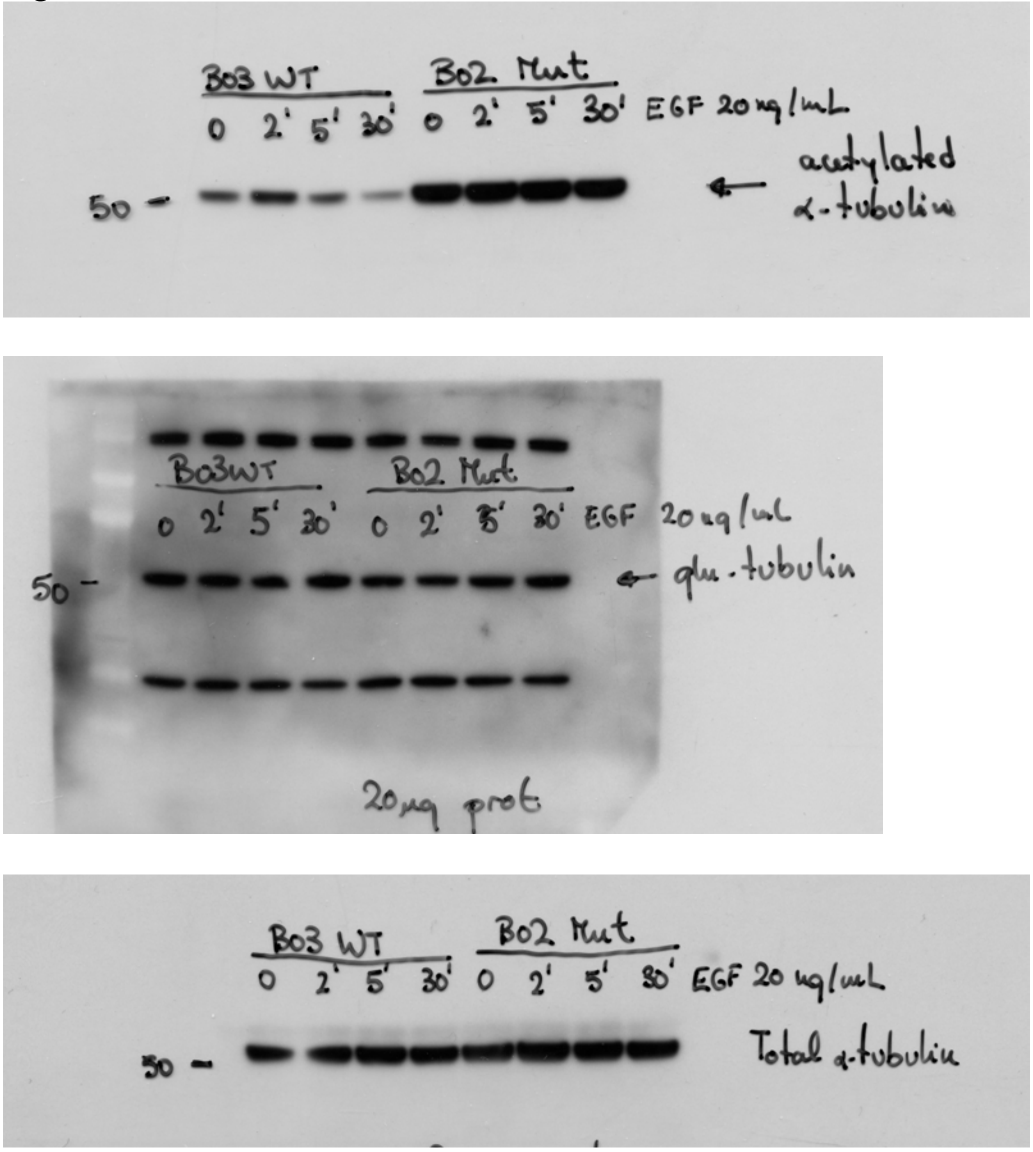

Figure S2C

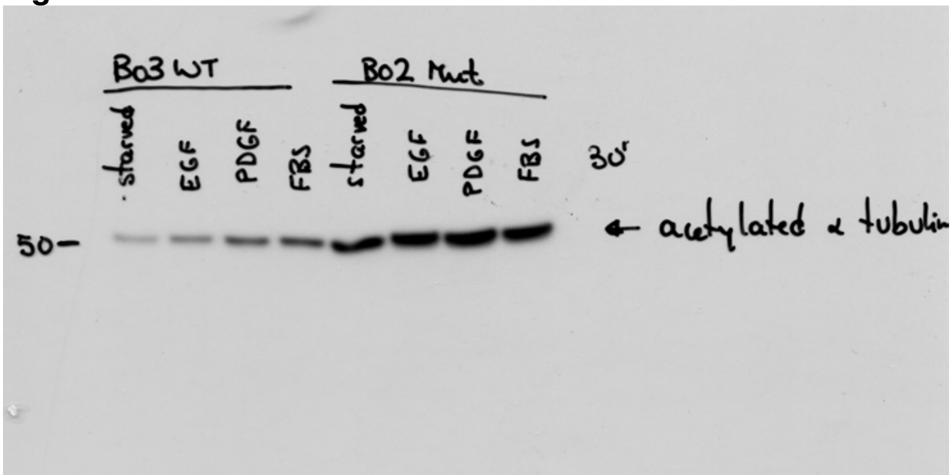

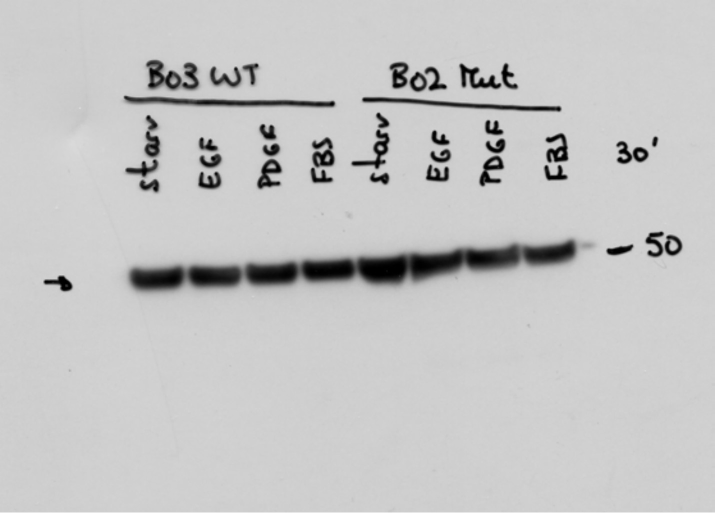

Figure S2D

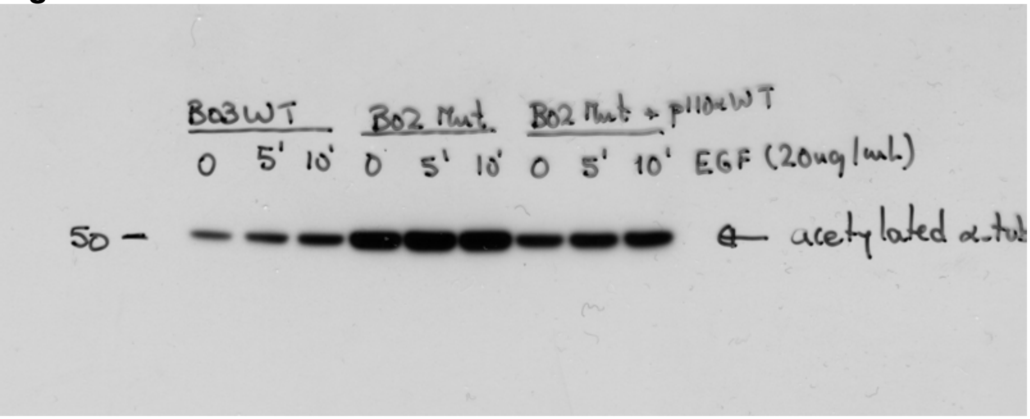

Figure S3F

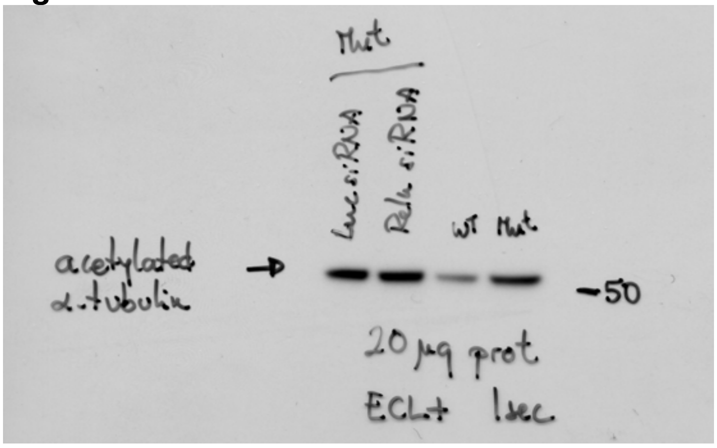

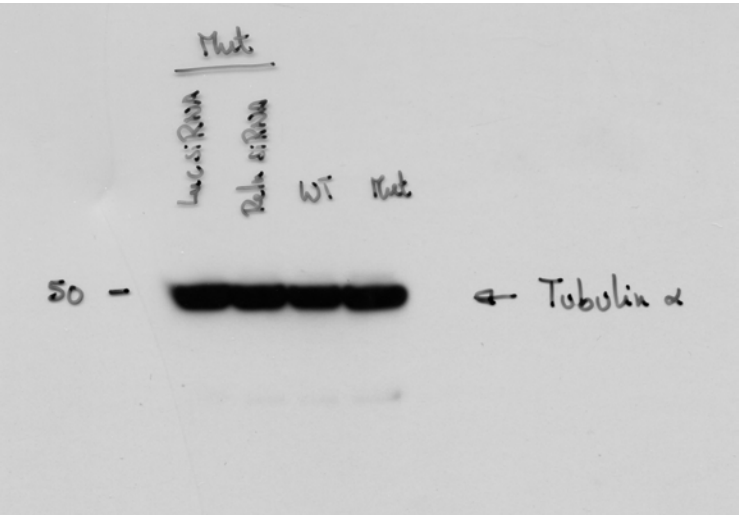

Figure S3G

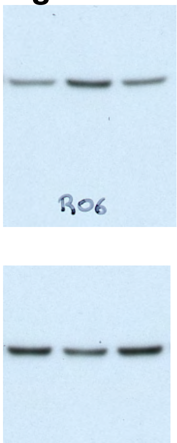

Supplement: Supplementary Information — Supplementary Figures 1-6 [file ncomms11245-s1.pdf]
